# The impact of temperature, humidity and closing school on the mumps epidemic: a case study in the mainland of China

**DOI:** 10.1186/s12889-024-18819-w

**Published:** 2024-06-19

**Authors:** Xiaoqun Li, Lianyun Zhang, Changlei Tan, Yan Wu, Ziheng Zhang, Juan Ding, Yong Li

**Affiliations:** 1 School of Information and Mathematics, Yangtze University, Nanhuan Road, Jingzhou, 434023 China; 2 Information Engineering College, Hunan Applied Technology University, Shanjuan Road, Changde, 415100 China; 3 Department of Operations Research and Information Engineering, Beijing University of Technology, Pingle Garden, Beijing, 100124 China; 4 School of Environment, Education & Development (SEED), The University of Manchester, Oxford Road, M139PL Manchester, UK; 5 Jingzhou Hospital Affiliated to Yangtze University, Chuyuan Avenue, Jingzhou, 434023 China

**Keywords:** Mumps, Basic reproduction number, Temperature, Humidity, School opening and closing, Data fitting

## Abstract

**Background:**

To control resurging infectious diseases like mumps, it is necessary to resort to effective control and preventive measures. These measures include increasing vaccine coverage, providing the community with advice on how to reduce exposure, and closing schools. To justify such intervention, it is important to understand how well each of these measures helps to limit transmission.

**Methods:**

In this paper, we propose a simple *SEILR *(susceptible-exposed-symptomatically infectious-asymptomatically infectious-recovered) model by using a novel transmission rate function to incorporate temperature, humidity, and closing school factors. This new transmission rate function allows us to verify the impact of each factor either separately or combined. Using reported mumps cases from 2004 to 2018 in the mainland of China, we perform data fitting and parameter estimation to evaluate the basic reproduction number ${\mathfrak R}_0$. As a wide range of one-dose measles, mumps, and rubella (MMR) vaccine programs in China started only in 2008, we use different vaccination proportions for the first Stage I period (from 2004 to 2008) and the second Stage II period (from 2009 to 2018). This allows us to verify the importance of higher vaccine coverage with a possible second dose of MMR vaccine.

**Results:**

We find that the basic reproduction number ${\mathfrak R}_0$ is generally between 1 and 3. We then use the Akaike Information Criteria to assess the extent to which each of the three factors contributed to the spread of mumps. The findings suggest that the impact of all three factors is substantial, with temperature having the most significant impact, followed by school opening and closing, and finally humidity.

**Conclusion:**

We conclude that the strategy of increasing vaccine coverage, changing micro-climate (temperature and humidity), and closing schools can greatly reduce mumps transmission.

**Supplementary Information:**

The online version contains supplementary material available at 10.1186/s12889-024-18819-w.

## Background

Mumps is an acute infection caused by the mumps virus (MuV). Mumps virus, parainfluenza, measles, respiratory syncytial virus and so on belong to paramyxoviruses, which are an RNA-type [1, 2]. The virus diameter is 85–300 nm, with an average of 140 nm [3, 4]. Mumps is a highly infectious disease, with more than 300,000 young people in China infected each year. Mumps can cause severe complications such as orchitis, oophoritis, pancreatitis, encephalitis, meningitis, and deafness [5–9]. Its primary route of early transmission is the virus carried by sneezing and coughing droplets of patients, inhaled, and deposited in the respiratory tract [3, 10]. Most patients are children and teenagers aged 2 and 24 with intense contact [5, 11–13]. The incubation period for mumps is about 15 to 24 days, of which 19 days is a median [3, 14, 15]. The most common strategy to prevent infection is mumps vaccination, which is usually part of a combination vaccine for measles, rubella, and mumps (MMR) [16, 17]. Many developed countries have adopted two doses of the mumps in their national immunization schedules. Though vaccinated people may still be infected with mumps, their risk for mumps has been reduced by about 78% for people who received one dose of MMR vaccine and about 88% for people who received two doses of MMR vaccine [9, 18, 19]. China started to introduce a dose of MMR vaccine to 18-month-old children in our free health insurance program in 2008 which has significantly reduced the mumps cases [2, 20].

Studies have found that the number of infectious diseases in the population changes periodically, and the incidence shows seasonal patterns [21–25], such as measles, chickenpox, rabies, influenza, etc. Like these infectious diseases, mumps cases showed strong seasonal patterns. Ukraine had more mumps cases in winter and spring [26]. Jordan also reported more mumps cases in winter and spring [27]. Mumps was found to have a significant peak in April in the United States [25]. In China, a significant peak was in April-July with another small peak in November and December [28, 29].

To have a better understanding of the spread and control of infectious diseases like mumps, mathematical models are commonly used to assess the impact of different factors including vaccine and population heterogeneity on the transmission of mumps. Qu et al. proposed a *SVEILHR* (*S*: susceptible, *V*: vaccinated, *E*: exposed, *I*: mild infectious, *L*: severe infectious, *H*: hospitalized, *R*: recovered) model with a seasonal varying transmission rate to simulate the seasonal outbreak of mumps, they found that improving vaccine coverage played an essential role in curbing the epidemics and recommended two doses of MMR vaccine in China [17]. Li et al. obtained the same finding from their *SVEILR* (*S*: susceptible, *V*: vaccinated, *E*: exposed, *I*: severely infectious, *L*: mildly infectious, *R*: recovered) model [16]. Nurbek et al. studied the effects of population heterogeneity and vaccine failure on mumps spread. They found that vaccine failure, changes in seasonality, and age structure were all associated with the mumps recurrence in Jiangsu Province [18]. Liu et al. also investigated the effects of population heterogeneity by constructing a multi-group *SVEIAR* (*S*: susceptible, *V*: vaccinated, *E*: exposed, *I*: symptomatically infected, *A*: asymptomatically infected, *R*: recovered) [30].

Rather than using a compartment model, a few researchers have used statistical regression models to analyze the association of temperature and humidity with the seasonal patterns of mumps [2, 7, 15, 28], where both temperature and humidity were considered independent factors. Hu et al. used a distributed hysteresis nonlinear model (DLNM) to evaluate the relationship between meteorological factors and the incidence of mumps in Fujian Province. They concluded that the lowest temperature and the highest relative humidity levels may increase mumps risk [2]. Yang et al. used the Poisson regression model combined with DLNM to evaluate the correlation of mumps incidence in Guangzhou, China, from 2005 to 2012 and concluded that the incidence of mumps increased with the increase of mean temperature and relative humidity [15]. Li et al. used the generalized additive model to quantify the relationship between meteorological factors and mumps in Jining, Shandong Province and concluded that the relationship between temperature and the incidence of mumps was J-shaped, with 4℃ corresponding to the minimum risk [28]. Ho et al. investigated the relationship between meteorological factors and the incidence of mumps in Taiwan, China, by using Poisson regression analysis and case-crossover and found that there was an inverted V-shaped relationship between the number of mumps cases and temperature. That is, the incidence of mumps began to rise when the temperature was 20℃ and began to decline again when the temperature was higher than about 25℃ [7].

In this paper, we propose a simple *SEILR* model by using a novel transmission rate function to incorporate temperature, humidity, and closing school. To our knowledge, this is the first paper to analyze the impact of all three factors together using a compartment dynamic model. There is a detailed description of our model in "Model building" section. He et al. used an *SIR* model to assess the seasonal patterns of the spread of influenza A in Canada [23] with a similar transmission rate $\beta\left(t\right)$ including temperature, humidity, and closing school factors.

The purpose of this study is to analyze the factors affecting mumps (temperature, humidity, and school opening and closing) based on a dynamic model and verify the applicability of the model by fitting the monthly case numbers of 31 districts, including 22 provinces, 5 autonomous regions, and 4 municipalities directly in the mainland of China (excluding Hong Kong, Macao, Taiwan) from 2004 to 2018, and finally calculate the basic reproduction number of each province, and then give some reliable measures. The structure of this article is as follows. In "Problem-driven and data-driven mumps model" section, we first introduce the area studied in this paper and the data sources needed for the research. Then, we establish the *SEILR* model and give the basic formula for calculating the basic reproduction number of this model. In "Methods" section, we focus on the methods used in this study, including data fitting methods, criteria for model selection, and methods for parameter sensitivity analysis. In "Results" section, we compare the ${\mathfrak R}_0$ in the three temperature zones, analyze the provinces with higher ${\mathfrak R}_0$, select the most appropriate model through the Akaike Information Criterion (*AIC*), explore the effects of temperature, humidity, and school opening and closing on the spread of mumps, interpret the parameters of the model, and analyze the sensitivities of the model parameters through the partial rank correlation coefficients (PRCC). In "Discussion" section, we recommend some preventive measures for controlling mumps epidemics, and conclude with a brief summary.

## Problem-driven and data-driven mumps model

### Study area

China (73°33′∼135°05′E, 3°51′∼53°33′N), with a land area of about 9.6 million square kilometers and a marine land area of about 4.73 million square kilometers [31], according to the results of China’s seventh population census, has about 1.443 billion people [32]. Its terrain and climate changes dramatically from the west to the east, as mumps cases are available only from the mainland of China, this study here excludes Hong Kong, Macao, and Taiwan.

**Fig. 1:**
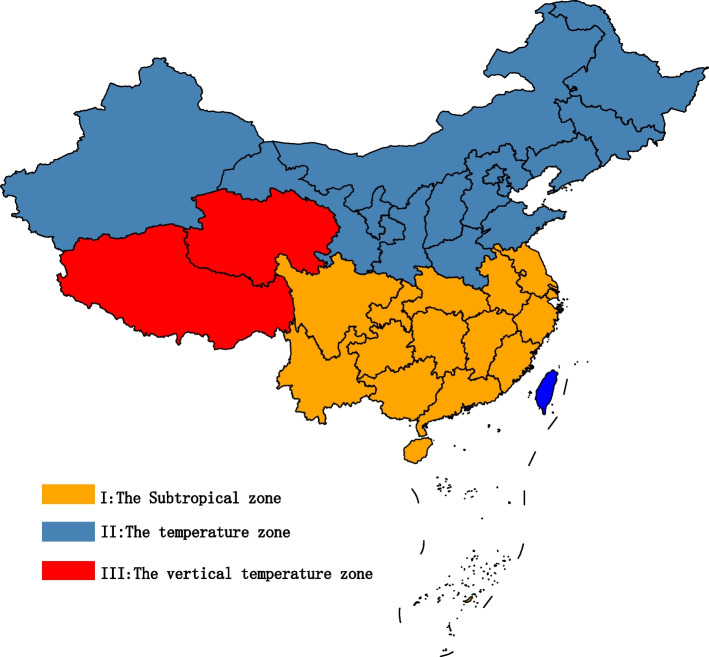
Three temperature zones of the mainland of China. ((I) Subtropical: Hainan, Guangdong, Guangxi, Yunnan, Jiangxi, Hunan, Chongqing, Guizhou, Fujian, Zhejiang, Hubei, Jiangsu, Sichuan, Anhui, Shanghai (Hainan is actually tropical, but because there is only one Hainan in the tropics, Hainan is classified as subtropical here for the convenience of discussion); (II) Temperate: Shandong, Beijing, Tianjin, Shanxi, Hebei, Henan, Shaanxi, Gansu, Xinjiang, Ningxia, Jilin, Heilongjiang, Liaoning, and Inner Mongolia; (III) The vertical temperature zone: Tibet and Qinghai.)

The Mainland of China has 31 districts, including 22 provinces, 5 autonomous regions, and 4 municipalities directly under the Central Government, based on their geographical location and climate [33], we group 31 districts into the following 3 temperate zones, see Fig. 1.

### Data sources

The study uses monthly mumps case data in the mainland of China during the 2004–2018 period. It is from the Public Health Science Data Center [4]. The monthly average temperature and humidity data is from the Chinese Bureau of Statistics [34]. Although each school semester’s opening and closing dates varied province by province, city by city, in general, the first semester of each year starts in September, and the winter vacation started in the middle of January of the following year. The second semester began in mid-February, and the summer vacation starts in July [35]. Here, schools include kindergarten, primary schools, middle schools, high schools, and universities to cover most mumps patients who were aged 2 to 24 years old in China.

### Model building

To make the model more effective, we make the following assumptions:

1. Assume that the populations we study are homogeneous, thus ignoring differences in gender, physical condition, etc., in each age group in each district.
2. According to relevant studies, mumps patients are predominantly adolescents, so our susceptible people only consider the proportion of the population aged 0–24 years. In this part, we obtain the annual population data of each district through the annual China Statistical Yearbook [34] and obtain the proportion of the population aged 0–24 years to obtain the relevant data of susceptible people (*S*) and the relevant data of overt patients (*I*) are obtained from Chinese Health Organizations.
3. Since the measles, rubella, and mumps vaccines are the same MMR vaccines, here we ignore the impact of other diseases and only consider mumps.

**Fig. 2:**
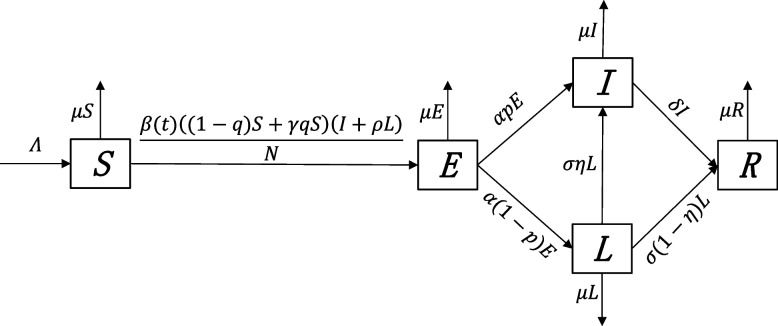
Flowchart of mumps transmission in a population

Our model is a simple *SEILR *(susceptible-exposed-symptomatically infectious- asymptomatically infectious-recovered). We divided the total population into five compartments: susceptible *S*, exposed *E*, symptomatic patients *I*, asymptomatic patients *L*, and recovered *R*. Total population *N* = *S* + *E* + *I* + *L* + *R*. A population size of $\Lambda$ enters the system, and the mortality rate of each compartment is specified as $\mu$. For the population of susceptible people (*S*), there are two ways of flow: one is death, and the other is the exposed (*E*) population with a transmission rate $\beta\left(t\right)$. There are two types of susceptible people (*S*): one is the vaccinated population, assuming its proportion is $q$; the other is the non-vaccinated population, then its proportion is (1*-q*), the infection capacity of vaccinated people is lower than that of non-vaccinated people, so we use $\gamma$ to represent the reduced ability of vaccinated individuals to become infected. There are two ways of flow for the exposed population ($E$): the first is to become a symptomatic patient ($I$), the second is to become an asymptomatic patient ($L$); we use $\alpha$ to present the rate of progression to infectious ($I$) per month. For this portion of the population, we use $p$ to represent the proportion of symptomatic patients ($I$). For symptomatic patients ($I$), their primary flow is recovery ($R$), where we use $\delta$ to represent the rate of symptomatic ($I$) to recovered patients ($R$) per month. There are two main flows of asymptomatic infected persons ($L$), where we denote by $\eta$ the rate of not symptomatic infected persons ($L$) to symptomatic infected persons ($I$), then ($1 - \eta$) persons flow to recovered persons ($R$), and we denote by $\sigma$ the rate of not symptomatic ($L$) to symptomatic ($I$) or recovered ($R$). The flowchart of this dynamics is shown in Fig. 2.

According to Fig. 2, we establish a system of ordinary differential equations to model the spread of mumps:

1 $$\left\{\begin{array}{l}\frac{dS}{dt}=\Lambda-\frac{\beta\left(t\right)\left(\left(1-q\right)S+\gamma qS\right)\left(I+\rho L\right)}N-\mu S,\\\frac{dE}{dt}=\frac{\beta\left(t\right)\left(\left(1-q\right)S+\gamma qS\right)\left(I+\rho L\right)}N-\alpha E-\mu E,\\\frac{dI}{dt}=\alpha\;pE-\delta I-\mu I+\sigma\eta L,\\\frac{dL}{dt}=\alpha\;\left(1-p\right)\;E-\sigma L-\mu L,\\\frac{dR}{dt}=\delta I+\sigma\left(1-\eta\right)\;L-\mu R.\end{array}\right.$$

As a wide range of one-dose free MMR vaccine programs in China started only in 2008 [2], we divide the study period into two stages to use different vaccination rates for each stage. Stage I: January 2004 to December 2008 has not yet been introduced. Stage II: January 2009 to December 2018. Vaccination rate $q$ is defined as follows:

2 $$q=\left\{\begin{array}{l}q_1,\;\mathrm{January}\;2004\;\mathrm{to}\;\mathrm{December}\;2008,\\q_2,\;\mathrm{January}\;2009\;\mathrm{to}\;\mathrm{December}\;2018.\end{array}\right.$$

The transmission rate $\beta \left( t \right)$ plays an essential role in the spread of mumps epidemics. As mumps outbreak shows strong seasonal patterns by following a school calendar, a number of researchers have used a simple sinusoidal function $\beta \left( t \right)$ = $\beta \left( 0 \right)$ + $\sin \left( {\omega t + \phi } \right)$ for the seasonal varying transmission rate [17, 18]. Rather than using a compartment model, a few researchers have used statistical regression models to analyze the association of temperature and humidity with the seasonal patterns of mumps [2, 15, 24, 28], where both temperature and humidity were considered independent factors.

Unlike previous studies, in this article, we will concretize $\beta \left( t \right)$ and consider the specific influencing factors on $\beta \left( t \right)$. To investigate the impact of temperature, humidity, and school opening and closing on the seasonal patterns of mumps cases in the mainland of China, we choose $\beta \left( t \right) = {\beta_0}{f_1}{f_2}{f_3}$. Where ${f_1}$ is a function of temperature, ${f_2}$ is a function of humidity, and ${f_3}$ is a function of school opening and closing.

Here,

3 $$\beta\left(t\right)=\beta_0f_1f_2f_3.$$

Now, we discuss how to choose functions $f_1,\;f_2$ and $f_3$ :

1) Temperature function $f_1$: Mumps is a seasonal infectious disease [25, 36, 37], and it is greatly affected by temperature. Relevant studies show that the number of cases of mumps will increase with the increase in temperature, and when it increases to a certain height, it will decrease with the increase in temperature. It becomes an inverted V-shape [7, 15]. So we introduce ${c_T}$ parameters as the optimal temperature, take the normal distribution function as the temperature function in this model.

4 $$f_1=\tau\frac1{\alpha_T\sqrt{2\pi}}\exp\left(-\frac{\left(T\left(t\right)\mathit-c_{\mathit T}\right)^2}{2{\mathrm\alpha}_{\mathrm T}^2}\right),$$

2) Humidity function$f_2$ : As humidity and temperature are strongly correlated to each other, Lin et al. showed that the number of mumps cases began to increase at a relative humidity of 65% to 69%. Several studies have also used exponential functions to explore the relationship between humidity and influenza transmission [24, 38, 39], so in this paper, we also assume to use an exponential function as the humidity function $f_2$ , with humidity as the independent variable.

5 $$f_2=e^{\alpha_h{H}(t)},$$

3) School vacation function $f_3$: In school vacations, students have less contact with other people, so its transmission rate should be smaller than the rate in school days [24]. Our data also show that the mumps cases decreased after school vacations started. Therefore, we use $\varepsilon$ as a reduction factor for the transmission of mumps during school winter vacation, and $\theta$ as a reduction factor for the transmission of mumps during school summer vacation. Now our school vacation function:

6 $$f_3=\left\{\begin{array}{ll}1,& \text{other time},\\1-\theta,& \text{summer time},\\1-\varepsilon,& \text{winter vacation}.\end{array}\right.$$

This completes the construct of time varying $\beta \left( t \right)$ with seasonal patterns. Table 1 summarizes all parameters in our model.

**Table 1:** The definition of parameters for the model (1)

Parameter	Definition
${q_1}$	Proportion of vaccination from January 2004 to December 2008
${q_2}$	Proportion of vaccination from January 2009 to December 2018
$\gamma$	Proportion of infection-reducing factor
$\rho$	Proportion of spread reduction factor
$\alpha$	Rate of progression to infectious per month
$p$	Proportion of infectious to symptomatic class
$\sigma$	Rate of not symptomatic to symptomatic or recovered
$\eta$	The proportion of not symptomatic to symptomatic per month
$\delta$	Rate of symptomatic to recovered patients per month
$\mu$	Natural death rate per month
$\Lambda$	New individuals coming into the system
$S$	The number of susceptible individuals
$E$	The number of exposed individuals
$I$	The number of infectious individuals symptomatic
$L$	The number of infectious individuals not symptomatic
$R$	The number of recovery individuals

### Calculation of basic reproduction number

As total population $N = S + E + I + L + R$, summarizing the left-hand side and right-hand side of the model (1) separately yields

7 $$\frac{dN}{{dt}} = \Lambda - \mu N.$$

The biologically feasible region of model (1) is

8 $$\Omega = \left\{ {\left( {S,E,I,L,R} \right) \in {\mathbb{R}}_+^5:0 \leqslant S + E + I + L + R \leqslant \frac{\Lambda }{\mu }} \right\}.$$

which can be verified as positively invariant (i.e., given non-negative initial values in $\Omega$, all solutions to model (1) have non-negative components and stay in $\Omega$ for $t \geqslant 0$) and globally attractive in ${\mathbb{R}}_+^5$ concerning model (1). Therefore, we restrict our attention to the dynamics of model (1).

It is easy to see that model (1) always has a disease-free equilibrium ${P_0}$,

9 $${P_0} = \left( {\frac{\Lambda }{\mu },0,0,0,0} \right).$$

By using the next-generation matrix and the concept of basic reproduction number [40, 41], we have the basic reproduction number, for details, see Appendix A.

10 $${R_0} = \frac{{\mathop {\beta (t)}\limits^\sim (\gamma q - q + 1)(\sigma \eta \alpha (1 - p) + \alpha p(\sigma + \mu ))}}{(\alpha + \mu )(\delta + \mu )(\sigma + \mu )} + \frac{{\mathop {\beta (t)}\limits^\sim \rho \alpha (1 - p)(\gamma q - q + 1)}}{(\alpha + \mu )(\sigma + \mu )}.$$

Of which,

11 $${{\mathop {\beta (t)}\limits^\sim}} = \frac{{\int_0^{T_0} {\beta (t)dt} }}{{T_0}},$$

where ${T_0}$ is the total time.

## Methods

### Data fitting

In this section, we first use model (1) to simulate the reported mumps data of most districts in the mainland of China, predict the disease trend, and obtain the influence of temperature, humidity, and school opening and closing on mumps. Data on mumps cases mainly come from the China Public Health Science Data Center [4]. We then must estimate the other 17 parameters and 5 initial values (See "Analysis of parameters" section for detailed ranges of parameters):

12 $$\varpi = (\alpha ,{\alpha_H},{\alpha_T},{\beta_0},\delta ,\eta ,\Lambda ,p,{q_1},{q_2},\gamma ,\rho ,\sigma ,\varepsilon ,\theta ,\tau ,{c_T},S(0),E(0),I(0),L(0),R(0)).$$

By calculating the minimum sum of the Chi-square error [42–44]:

13 $$H(\varpi ) = \sum\limits_{i = 1}^n {\frac{{(I({t_i}) - \mathop {I({t_i}}\limits^\sim ){)^2}}}{{\mathop {I({t_i})}\limits^\sim }}. }$$

Where $n$ represents the total number of months of simulation time in each province, $I\left( {t_i} \right)$, $i = 1,2,3, \cdots ,n$ represents the true number of cases per month. $\mathop {I({t_i}}\limits^\sim )$, $i = 1,2,3, \cdots ,n$ represents the fitted value of monthly cases. MATLAB tool Particle swarm function [45–47] is used to solve the multidimensional unconstrained linear optimization problem, and the minimum value of the multivariable unconstrained function $H\left( \varpi \right)$ is found by the derivative-free method to determine the optimal parameter value $\varpi$. The data fitting for each district is shown in Supplementary Information.

### Selection criteria for epidemiological models

In epidemiological modeling studies, there are usually criteria to explore the plausibility of the models. Among them, the Akaike Information Criterion ($AIC$) and Bayesian Information Criterion ($BIC$) are the two most commonly used methods to measure the goodness-of-fit and complexity of statistical models [48, 49]. Although $AIC$ and $BIC$ are tools for choosing between different models, the results of the two criteria for the same model may differ due to their trade-offs and the degree of penalty for complexity. $BIC$ penalizes model complexity more strictly and is sensitive to sample size, so it is generally used to select simple models with fewer parameters. $AIC$ punishes model complexity more leniently and is not particularly sensitive to sample size. That is, it can tolerate the complexity of the model to a certain extent, so it is generally used in situations where the sample is relatively large. Our model has a large sample size and is more suitable for $AIC$ [50]. This criterion measures the relative goodness-of-fit of a mathematical model, penalizes overfitting, encourages the selection of models that fit well with the data, and compares the strengths and weaknesses of multiple competing models. However, if you want the number of parameters of the fitted mathematical model to include a more significant penalty, you need to use the $AIC$ version by modification, also known as $AICc$ [51–53]. At the same time, since $AIC$ and $AICc$ are of an arbitrary scale and difficult to explain, it can also be obtained that different competing models have relative support in the same data by calculating the value of ${\Delta_j}$ [52]. Another helpful way to measure a model’s support for data is the Akaike weight ${\omega_i}$ [52]. Based on these information criteria, a model is given:

14 $$AIC = n\ [\ln (\frac{SSE}{n})] + 2k,$$

15 $$AICc = n\ [\ln (\frac{SSE}{n})] + 2k + \frac{2k(k + 1)}{{n - k - 1}},$$

16 $${\Delta_j} = AI{C_j} - AI{C_{\min }},$$

17 $${\omega_i} = \frac{{{e^{ - \frac{{\Delta_i}}{2}}}}}{{\sum\limits_{j = 1}^J {{e^{ - \frac{{\Delta_j}}{2}}}} }}.$$

Where $n$ represents the number of data points in the dataset, $k$ represents the number of fitted parameters plus one, and $SSE$ is the least squares error. $AI{C_j}$ is the $AIC$ of the $jth$ model, and $AI{C_{\min }}$ is the optimal $AIC$ model. ${\Delta_j}$ represents the difference between the $AIC$ value of ${\text{mode}}{{\text{l}}_j}$ and the $AIC$ value of the optimal model. ${\omega_i}$ represents the weight of ${\text{mode}}{{\text{l}}_i}$, measuring the relative contribution of each model to data interpretation. When the $SSE$ is smaller and the $AIC$ is smaller, the better the $AICc$ model fits. The smaller ${\Delta_j}$, the closer the surface ${\text{mode}}{{\text{l}}_j}$ is to the optimal model on the fitted data. The criteria for ${\Delta_j}$ are shown in Table 2. For the Akaike weight ${\omega_i}$, if the Akaike weight ${\omega_i}$ of the fitted model is more significant than other models, the model is relatively supported in the data.

**Table 2:** The criteria of ${\Delta_j}$ for model selection

${\Delta_j}$ for model selection	Support power
${\Delta_j} \leqslant 2$	substantial support
$4 \leqslant {\Delta_j} \leqslant 7$	considerably less support
${\Delta_j} > 10$	essentially no support

### Partial rank correlation coefficients

Sensitivity analysis (SA) is a method to identify and quantify the effect of parameter uncertainty on the basic reproduction number ${\Re_0}$. The model’s predictability is improved by controlling the critical parameters identified that significantly impact the model output. Here, we assume that each parameter is a random variable with a uniform distribution and analyze the model’s sensitivity through the uncertainty of Latin hypercube sampling. At present, this method has been applied to many epidemiological models [54, 55].

We first use Latin hypercube sampling to sample the parameters that appear in the basic reproduction number ${\Re_0}$, and then calculate the partial rank correlation coefficients (PRCC) based on the LHS matrix to analyze the sensitivity of the parameters to ${\Re_0}$ and the model to determine the extent and way these parameters affect ${\Re_0}$. Therefore, to examine the sensitivity of ${\Re_0}$ when the parameter changes, we use Latin hypercube sampling to examine the dependence of the parameters on ${\Re_0}$.

## Results

### Data fitting and model selection

**Fig. 3:**
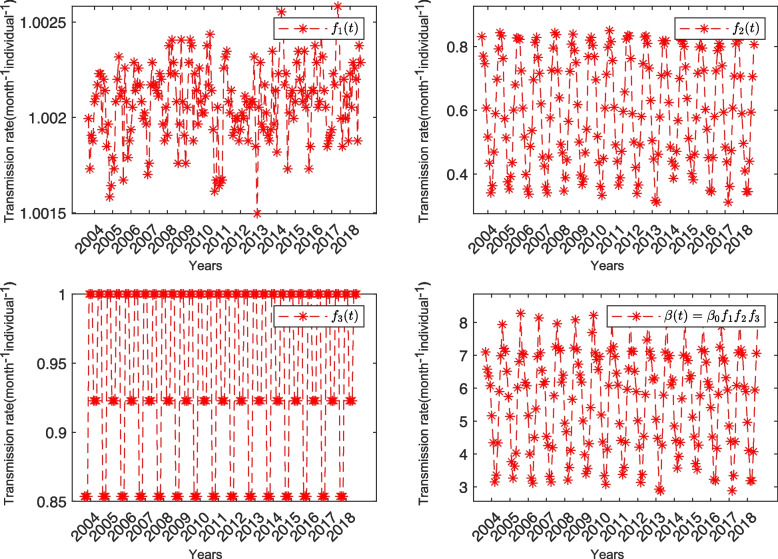
Temperature function ${f_1}\left( t \right)$, the relative humidity function ${f_2}\left( t \right)$, the school opening and closing function ${f_3}\left( t \right)$, and the transmission rate function $\beta \left( t \right)$ in the model ${U_1}$

To explore the degree of influence of temperature, humidity, and closing school from a mechanical perspective, we have taken into account the following sub-models: (${U_1}$) All three factors of temperature, humidity, and closing school are taken into account ($\beta \left( t \right) = {\beta_0}{f_1}{f_2}{f_3}$). (${U_2}$) The temperature factor is removed (${f_1} = 1$). (${U_3}$) The humidity factor is removed (${f_2} = 1$). (${U_4}$) The school opening and closing factor is removed (${f_3} = 1$). Here, we only select the reported cases in Jiangsu Province for discussion, and the discussion in other provinces is similar, and a plot of the effects of ${f_1}$, ${f_2}$, and ${f_3}$ can be seen in Fig. 3.

**Fig. 4:**
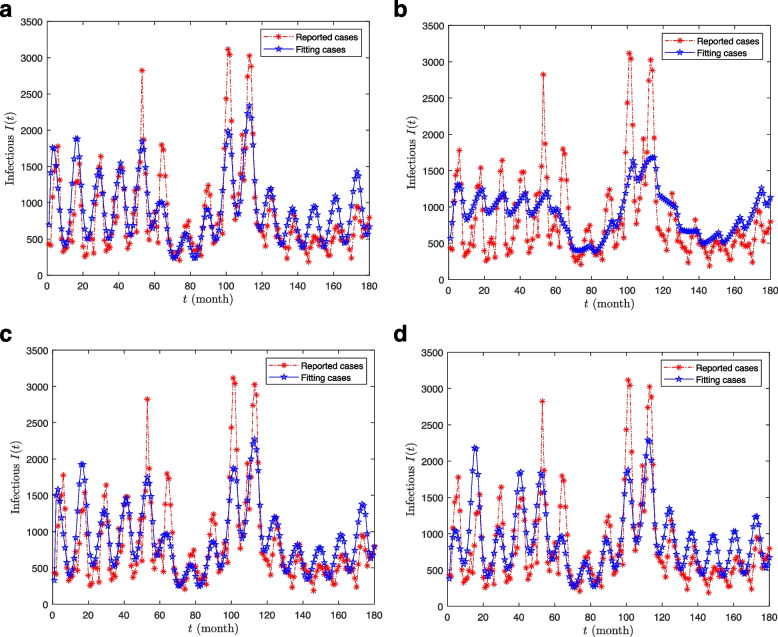
The fitting effects of four models (${U_1}$-${U_4}$)

We want to select the model from four groups of models (${U_1}$) -(${U_4}$) that best describe the fitting effect of the data based on the method of judging the strength of the model (The fit of the four sets of models can be seen in Fig. 4).

Based on the Akaike Information Criterion ($AIC$), Table 3 shows that $SSE$, $AIC$, and $AICc$ of model ${U_1}$ are the smallest in ${U_1} - {U_4}$, and ${\Delta_1} < 2$, that is to say, when all three factors are considered, the data support for the model is the highest, ${U_3}$ has relatively little support, while ${U_2}$ and ${U_4}$ are essentially unsupported relative to each other. At the same time, ${\omega_1} > {\omega_3} > {\omega_4} > {\omega_2}$, it can also be seen that the impact of these three factors on the degree of mumps from large to small in the order of temperature, school opening and closing, and humidity.

**Table 3:** The value of selection criteria for model ${U_1} - {U_4}$

Model	Fitted parameters	$SSE$	$AIC$	$AICc$	$\Delta AIC$	${\omega_i}$
${U_1}$	17 param	$2.28 \times {10^7}$	$2150.95$	$2155.19$	0	$0.7908$
${U_2}$	14 param	$3.63 \times {10^7}$	$2228.9$	$2231.92$	$76.73$	$1.7234 \times {10^{ - 17}}$
${U_3}$	15 param	$2.35 \times {10^7}$	$2154.07$	$2157.85$	$2.66$	$0.2092$
${U_4}$	16 param	$2.911 \times {10^7}$	$2190.86$	$2194.2$	$39.01$	$2.6741 \times {10^{ - 7}}$

### Analysis of parameters

We analyze the parameters of the model ${U_1}$, some of which have been estimated by WHO, some of which have been evaluated by mumps researchers and predecessors, and others which remain uncertain. The following parameters are analyzed in detail (The data for these parameters can be detailed in Table 4).

**Table 4:** The values and sources of the parameters and calculated ${\Re_0}$ for the models ${U_1} - {U_4}$

Parameter	${U_1}$	${U_2}$	${U_3}$	${U_4}$	Source
$\Lambda$	8275	41,014	10,560	77,530	Estimate
$S(0)$	15,169	32,866	10,280	10,000	Estimate
$E(0)$	10,000	15,942	10,004	30,416	Estimate
$I(0)$	698	566	330	381	Estimate
$L(0)$	637	885	30	6741	Estimate
$R(0)$	8702	3498	4459	155	Estimate
${\beta_0}$	9.992	9.1173	7.649	5.173	Estimate
$\alpha$	1.3063	1.7489	2	1.3165	Estimate
$p$	0.0563	0.035	0.0944	0.0675	Estimate
$\delta$	1.5356	2.0133	2.9938	2.8565	Estimate
$\sigma$	2.1093	1.9656	1.7878	2.5362	Estimate
$\eta$	0.2575	0.0597	0.4746	0.0036	Estimate
${q_1}$	0.1935	0.3609	0.3088	0.177	Estimate
${q_2}$	0.8395	0.867	0.9997	0.6358	Estimate
$\gamma$	0.7718	0.7836	0.851	0.8304	Estimate
$\rho$	0.7199	0.4803	0.7926	0.9242	Estimate
$\mu$	0.0011	0.0011	0.0011	0.0011	Fix
${\Re_0}(stage I)$	2.5157	2.3642	2.2066	2.1516	Calculate
${\Re_0}(stage II)$	2.1463	2.0834	1.9773	1.9625	Calculate
${\Re_0}$	2.2695	2.177	2.0537	2.0255	Calculate

1) The natural mortality rate $\mu$ is numerically equal to the inverse of life expectancy at birth. The average age from 2004 to 2018 is 76.63 [34], i.e., $\mu$ = 1/(76.63$\times$ 12), therefore $\mu$ = 0.0011.

2) The rate of monthly conversion from exposed ($$E$$) to the patient ($$I$$ and $$L$$) $$\alpha$$: the incubation period of mumps is usually 15–24 days [14, 15], we studied monthly data, and the reciprocal of $$\alpha$$ indicates the incubation period, so we assume $$\alpha\;\in\;\lbrack1.25,\;2\rbrack$$, according to parameter estimation $$\alpha = 1.3063$$, the incubation period of mumps is $${\raise0.7ex\hbox{${30}$} \!\mathord{\left/ {\vphantom {{30} \alpha }}\right.\kern-0pt}\!\lower0.7ex\hbox{$\alpha $}} = 23$$ days.

3) The proportion of monthly conversion from exposed patients ($E$) to symptomatic patients ($I$) $p$: we set $p\in\left[0,1\right]$, according to parameter estimation $p = 0.0563$, about $5.63\%$ of exposed patients will turn into symptomatic patients every month, and about. $1 - p = 94.37\%$ of exposed patients will turn into asymptomatic patients every month.

4) Rate of symptomatic ($$I$$) to recovered patients ($$R$$) per month $$\delta$$: symptomatic patients usually recover in about 10 days, considering that we study monthly case data from 31 districts in the mainland of China for a total of 180 months from 2004–2018, which involves a large range of recovery time of 7–30 days, so we set $$\delta\in\lbrack1,\ {\raise0.7ex\hbox{${30}$} \!\mathord{\left/ {\vphantom {{30} 7}}\right.\kern-0pt}\!\lower0.7ex\hbox{$7$}}]$$.

5) Rate of progression from asymptomatic patients ($$L$$) to symptomatic patients ($$I$$) or recovered patients ($$R$$) $$\sigma$$: similar to $$\delta$$, we also set $$\sigma\;\in\;\lbrack1,30/7\rbrack$$, according to the parameter estimate $$\sigma = 2.1093$$, the asymptomatic patients ($$L$$) will become symptomatic patients ($$I$$) or recovered patients ($$R$$) after about $${\raise0.7ex\hbox{${30}$} \!\mathord{\left/ {\vphantom {{30} \sigma }}\right.\kern-0pt}\!\lower0.7ex\hbox{$\sigma $}} = 14$$ days [56].

6) The proportion of asymptomatic patients ($L$) transformed into symptomatic patients ($I$) $\eta$: most asymptomatic patients with mumps will recover [56], thus, we set $\eta \in [0,0.5]$. According to the parameter estimation $\eta = 0.2575$, it can be seen that about $25.75\%$ of asymptomatic patients are transformed into patients every month, and $1 - 25.75\% = 74.25\%$ of asymptomatic patients will recover.

7) Vaccine coverage rate $q$, we make the following subsections: 2004–2008 vaccine is not free, the vaccination situation in this period is not clear, so we set $q_1\;\in\;\lbrack0.01,1\rbrack$, 2009–2018 this period China will be the mumps vaccine into the relevant health insurance plan [2, 20], according to China’s relevant school enrollment policy [57], school children must be vaccinated against relevant vaccines, including the mumps vaccine, through China’s population data we find that the age of the population of 0–12 years old is at least 20% [34], so we set $q_2\in\lbrack0.2,1\rbrack$. According to the parameter estimation, ${q_1} = 0.1935$ and ${q_2} = 0.8395$, it can be concluded that after the popularization of MMR vaccine in China in 2008, the vaccination rate has significantly increased, and the vaccination rate also has a specific effect on the prevention and control of mumps.

8) $\gamma$ and $\rho$ denote the infection reduction factor and propagation reduction factor respectively. Cases that can be diagnosed and categorized by laboratory pathology are unknown, so we set $\gamma , \rho \in [0,1]$.

### Parameter sensitivity analysis

**Fig. 5:**
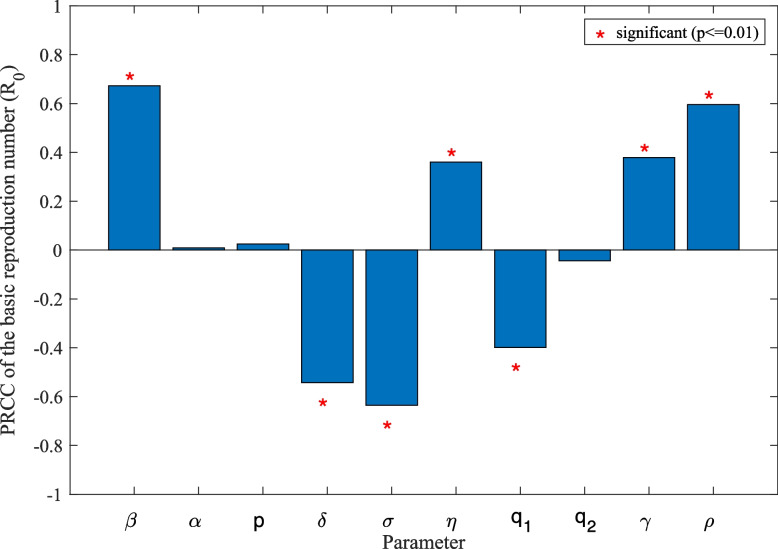
Show the PRCC of parameters with ${\Re_0}$

**Fig. 6:**
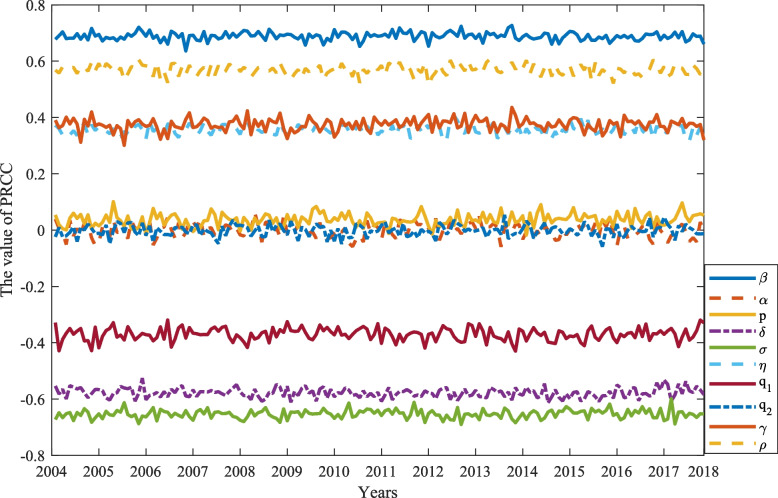
PRCC of parameters in continuous time

We set the sample volume to $n = 2000$ and take the parameters in the analog as an input variable and the value of ${\Re_0}$ as an output variable. The PRCC on the 10 parameters is shown in Fig. 5. Among them, the effect of parameters on the result is mainly reflected in the absolute value of PRCC values for ${\Re_0}$ can be seen on Table 5. The greater the absolute value of the PRCC of the parameter, the more significant its impact on the change of ${\Re_0}$, and the positive or negative influence is positive or negative. In our experiments, we assume that the parameters of the p-value less than or equal to 0.01 have a significant impact, at the same time, focus on analyzing the parameters of the absolute value of its PRCC > 0.2. We can easily see that different parameters affect ${\Re_0}$. Among them, $\beta , \eta , \gamma , \rho$ significantly actively affect ${\Re_0}$, while $\delta , \sigma$, and ${q_1}$ significantly negatively affect ${\Re_0}$. Figure 5 shows that the contact rate $\beta$ (PRCC = 0.6727) has the most significant effect on ${\Re_0}$, then followed by the rate of not symptomatic to symptomatic or recovered per month $\sigma$ (PRCC = -0.6349) and by the rate of symptomatic to recovered patients per month $\delta$ (PRCC = -0.5430). Meanwhile, Fig. 6 shows that we also analyze PRCC of every parameter over continuous time. In "Discussion" section, we will analyze the parameters that have a more significant impact by the sensitivity analysis and propose some measures to cope with mumps.

**Table 5:** Partial rank correlation coefficients (PRCC) values for ${\Re_0}$

Input parameter	$\beta$	$\alpha$	$p$	$\delta$	$\sigma$
PRCC	0.6727	0.0095	0.0247	-0.5430	-0.6349
*p*-value	$1.70 \times {10^{ - 262}}$	0.6727	0.2701	$4.54 \times {10^{ - 153}}$	$4.16 \times {10^{ - 225}}$
Input parameter	$\eta$	${q_1}$	${q_2}$	$\gamma$	$\rho$
PRCC	0.3606	-0.3990	-0.0437	0.3792	0.5957
*p*-value	$3.28 \times {10^{ - 62}}$	$5.82 \times {10^{ - 77}}$	0.0512	$4.24 \times {10^{ - 69}}$	$1.54 \times {10^{ - 191}}$

### Comparative analysis of ${\Re_0}$

**Fig. 7:**
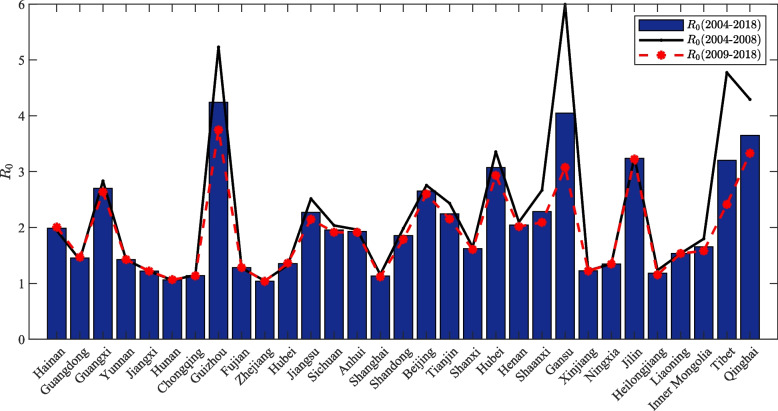
The value of ${\Re_0}$ for each province in the mainland of China

The respective temperature and humidity of Zone I-III are shown in Table 6. And the basic reproduction number ${\Re_0}$ for the 31 districts in the mainland of China is shown in Fig. 7. It is found fitting that ${\Re_0}$ of subtropical has roughly distributed around 1–3. It should be noted that ${\Re_0}$ in Guizhou Province is relatively high, and its value is 4.2413. Zhang et al. proposed in the relationship between meteorological factors and mumps based on the Boosted regression tree model that with the increase of temperature, the occurrence of mumps shows an upward trend, and 4℃ corresponds to the minimum risk [5]. Hence, the lower the temperature below 4℃, the greater the ${\Re_0}$; the higher the temperature above 4℃, the more likely the ${\Re_0}$ is to be larger. While the lowest temperature in Guizhou Province is -1.5℃, the maximum temperature is 24.3℃, and the average temperature is 14.67℃, we consider that ${\Re_0}$ in Guizhou Province is affected by temperature. Yang et al. proposed that the relationship between meteorological factors and mumps incidence in Guangzhou from 2005 to 2012 that the higher the relative humidity [15], the greater ${\Re_0}$ of mumps, while the lowest relative humidity in Guizhou Province is 0.65, the highest relative humidity is 0.92, the average relative humidity is 0.79. It can be found that the relative humidity in Guizhou is still relatively high. We consider that ${\Re_0}$ of Guizhou is greatly affected by relative humidity. Therefore, ${\Re_0}$ in Guizhou may be related to the low temperature, humidity, and vaccination rate in Guizhou. ${\Re_0}$ in temperate regions is also roughly around 1–3, and it should also be noted that the basic reproduction number ${\Re_0}$ in Gansu Province is slightly larger, is 4.0485. Check the data to discover the particularity of the natural environment of Gansu Province. Gansu Province has a subtropical monsoon climate, temperate monsoon climate, temperate continental (arid) climate, plateau alpine climate, and four other major climate types. Hamami et al., in studying weakened immunity and mumps outbreaks, concluded that the weakening of human immunity is the main factor in the outbreak of various epidemics [58], so here we consider that mumps in Gansu Province is not only related to temperature, humidity, school holidays, etc. but may also be related to its complex climate type and low vaccination rate. ${\Re_0}$ is about 3 in the vertical temperature zone, which is relatively consistent.

**Table 6:** Temperature and humidity of the three temperature zones (AM: Arithmetic Mean; SD: Standard Deviation)

Zone	Temperature(°*C*)				Humidity(%)			
	Interval	AM	Median	SD	Interval	AM	Median	SD
I	[-21.1, 30.5]	10.9	13	11.9	[0.22, 0.89]	0.57	0.57	0.13
II	[-1.5, 32.6]	18.5	19.4	7.8	[0.42, 0.94]	0.74	0.75	0.08
III	[-11.2, 20.2]	7.8	8.8	7.9	[0.14, 0.77]	0.47	0.48	0.16

Through calculations, we found that the average ${\Re_0}$ in the subtropical region is 1.7126, which is relatively the smallest; the average ${\Re_0}$ in the temperate region is 2.1429, which is in the middle position; the average value of ${\Re_0}$ in the vertical temperature zone of the Qinghai-Tibet Plateau is 3.4252, which is relatively the largest. Considering its relationship with temperature, we guess that the mumps virus activity may be more potent at low temperature, which is also the problem we will explore later. For threshold systems, if ${\Re_0}$ < 1, mumps can be eliminated, and the disease can be controlled. If ${\Re_0}$ > 1, the condition will remain at the epidemic level, and specific isolation measures need to be taken to control the spread of mumps effectively. In the Netherlands, Wales, and England, ${\Re_0}$ of mumps was about 11–14 from 1960 to 1980, ${\Re_0}$ by the state in the United States is roughly distributed in 4–7 [59]. However, ${\Re_0}$ of mumps in China is relatively small [59]. By consulting the relevant literature, ${\Re_0}$ of mumps in China in recent years is estimated to be about 6.5428 [17]. Li et al. estimated the prevalence of mumps in the mainland of China at 4.28 [16], and Nurbek et al. estimated the prevalence of mumps in Jiangsu Province from 2005 to 2019 at 1–2 [18]. There may be some variation in the results of ${\Re_0}$ in each country and region due to the influencing factors studied in the selected case data. ${\Re_0}$ of mumps in the mainland of China learned here was estimated to be 4.5933. It can be seen that mumps has been better controlled than before. However, the ${\Re_0}$ > 1 here indicates that mumps is still at the epidemic level, so isolation, prevention, and control still need to be strengthened.

## Discussion

The findings in this study show that vaccination is still one of the most effective strategies to prevent mumps epidemics, as ${\Re_0}$ (Stage I) > ${\Re_0}$ (Stage II). For most provinces and cities in China, only one dose of free MMR vaccine is available, while the most developed countries have implemented two doses of MMR vaccine program. The American Academy of Pediatrics recommends that children receive the first dose of the MMR vaccine at 12 to 15 months of age and the second dose is at 4 to 6 years of age [6]. Since the 1980s, most European countries have routinely immunized children against mumps [60]. Therefore, China should maintain the current one dose of MMR vaccine program and encourage more children to receive a second dose. We fit the data for each of the 31 districts and calculate ${\Re_0}$, and find that ${\Re_0}$ for most districts are concentrated in 1–3. We also get the basic reproduction number for the three temperature zones and find that the ${\Re_0}$ of the Qinghai-Tibet Plateau > middle temperate zone ${\Re_0}$ > subtropical zone ${\Re_0}$, which clearly shows that the temperature and humidity have an impact on the spread of mumps.

We use Akaike Information Criteria to evaluate the four models ${U_1}$-${U_4}$ and find that temperature has the most significant effect on mumps, followed by closing school, and humidity has the lowest impact on mumps epidemics.

Our results show that mumps is a resurging disease. The three parameters $\beta , \delta$, and $\sigma$ have the greatest influence on ${\Re_0}$. We can reduce ${\Re_0}$ by decreasing $\beta$ and increasing $\delta$ and $\sigma$ so that we can prevent mumps. $\beta \left( t \right)$ is related to temperature, humidity, and the closing school. We suggest that in the early stage of mumps outbreaks, it can be prevented by increasing vaccination, personal hygiene, and closing schools; at the peak of mumps outbreaks, attention should be paid to the effects of temperature and humidity on mumps; when the temperature is too high, we can help cool it down by sprinkler watering and planting greenery; when the humidity is too high, attention should be paid to the opening of windows to ventilate the room and air conditioning and dehumidifiers can be used to lower the humidity; in the peak of mumps outbreaks, attention should also be given to the contact among students, and certain isolation measures should be taken if necessary; and when the peak is over, disinfection should be paid attention to, and attention should be paid to personal protection. $\delta$ denotes the rate of symptomatic patients to recovery and $\sigma$ denotes the recovery rate of asymptomatic patients in this paper, an effective measure to increase $\delta$ and $\sigma$ is to strengthen the immune system, which can be achieved through vaccination and physical exercise. So we recommend strengthening the second dose of mumps vaccination, and more importantly, educating raising awareness of the preparedness to deal with mumps, and if necessary, isolation measures to deal with mumps [61].

Our findings show that mumps is still a disease of great impact in China. More importantly, mumps control measures should be intensified in the high-risk areas of the vertical temperature zone including the Qinghai-Tibet Plateau, especially, in the school term periods. Apart from increasing vaccination coverage, we only study the impact of three factors of temperature, humidity, and closing schools on the mumps in the mainland of China, other factors like social economics, public health resources, and population heterogeneity may also have a social economic impact on mumps outbreaks, we will investigate these factors in future.

### Supplementary Information

Supplementary Material 1.

## Data Availability

No datasets were generated or analysed during the current study.

## Appendix A: The detailed calculation process of ${\Re_0}$

Following the discussion in Sect. "Calculation of basic reproduction number", model (1) always has a disease-free equilibrium ${P_0}$ and ${P_0}$ is the solution of the algebraic equations:

18 $$\left\{ \begin{gathered} \Lambda - \frac{{\beta \left( t \right)\left( {\left( {1 - q} \right)S + \gamma qS} \right)\left( {I + \rho L} \right)}}{N} - \mu S = 0, \hfill \\ \frac{{\beta \left( t \right)\left( {\left( {1 - q} \right)S + \gamma qS} \right)\left( {I + \rho L} \right)}}{N} - \alpha E - \mu E = 0, \hfill \\ \alpha pE - \delta I - \mu I + \sigma \eta L = 0, \hfill \\ \alpha \left( {1 - p} \right)E - \sigma L - \mu L = 0, \hfill \\ \delta I + \sigma \left( {1 - \eta } \right)L - \mu R = 0, \hfill \\ I = 0. \hfill \\ \end{gathered} \right. \,$$

And

$${P_0} = \left( {\frac{\Lambda }{\mu },0,0,0,0} \right).$$

We use the next generation matrix to derive basic reproduction number [40, 41]. First, we change the order of model (1) by

$${\left( {\frac{dE}{{dt}},\frac{dI}{{dt}},\frac{dL}{{dt}},\frac{dR}{{dt}},\frac{dS}{{dt}}} \right)^T} = F - V .$$

Let

19 $$\mathop {\beta (t)}\limits^\sim = \frac{{\int_0^{T_0} {\beta (t)dt} }}{{T_0}},$$

Here,

$$\mathfrak{F}{ = }\left( {\begin{array}{*{20}{c}} {\frac{{\mathop {\beta \left( t \right)}\limits^\sim \left( {\left( {1 - q} \right)S + \gamma qS} \right)\left( {I + \rho L} \right)}}{N}} \\ 0 \\ 0 \\ 0 \\ 0 \end{array}} \right).$$

and

$$\mathfrak{V}{ = }\left( {\begin{array}{*{20}{c}} {\alpha E{ + }\mu {\rm E}} \\ { - \alpha pE + \delta I + \mu I - \sigma \eta L} \\ { - \alpha \left( {1 - p} \right)E + \sigma L + \mu L} \\ { - \delta I - \sigma (1 - \eta )L + \mu R} \\ { - \Lambda + \frac{{\mathop {\beta \left( t \right)}\limits^\sim \left( {\left( {1 - q} \right)S + \gamma qS} \right)\left( {I + \rho L} \right)}}{N} + \mu S} \end{array}} \right).$$

The Jacobian matrix F and V of $\mathfrak{F}$ and $\mathfrak{V}$ at ${P_0}$ are:

$$\begin{gathered} F = \frac{{\partial\mathfrak{F}\left( {E,I,L,R,S} \right)}}{{\partial \left( {E,I,L,R,S} \right)}}\left| \begin{gathered} \hfill \\ {P_0} \hfill \\ \end{gathered} \right. \hfill \\ = \left( {\begin{array}{*{20}{c}} 0&{\mathop {\beta (t)}\limits^\sim \left( {\left( {1 - q} \right) + \gamma q} \right)}&{\mathop {\beta (t)}\limits^\sim \rho \left( {\left( {1 - q} \right) + \gamma q} \right)}&0&0 \\ 0&0&0&0&0 \\ 0&0&0&0&0 \\ 0&0&0&0&0 \\ 0&0&0&0&0 \end{array}} \right). \hfill \\ \end{gathered}$$

and

$$\begin{gathered} V = \frac{{\partial\mathfrak{V}\left( {E,I,L,R,S} \right)}}{{\partial \left( {E,I,L,R,S} \right)}}\left| \begin{gathered} \hfill \\ {P_0} \hfill \\ \end{gathered} \right. \hfill \\ = \left( {\begin{array}{*{20}{c}} {\alpha { + }\mu }&0&0&0&0 \\ { - \alpha p}&{\delta { + }\mu }&{ - \sigma \eta }&0&0 \\ { - \alpha \left( {1 - p} \right)}&0&{\sigma { + }\mu }&0&0 \\ 0&{ - \delta }&{ - \sigma (1 - \eta )}&\mu &0 \\ 0&{ \, \mathop {\beta (t)}\limits^\sim \left( {\left( {1 - q} \right) + \gamma q} \right)}&{\mathop {\beta (t)}\limits^\sim \rho \left( {\left( {1 - q} \right) + \gamma q} \right)}&0&\mu \end{array}} \right). \hfill \\ \end{gathered}$$

the inverse of $V$ is

$${V^{ - 1}} = \left( {\begin{array}{*{20}{c}} {\frac{1}{\alpha + \mu }}&0&0&0&0 \\ {\frac{{\alpha {A_4}}}{M}}&{\frac{1}{\delta + \mu } \, }&{\frac{{\eta \sigma \left( {\alpha + \mu } \right)}}{M}}&0&0 \\ { - \frac{{\alpha \left( {p - 1} \right)(\delta + \mu )}}{M}}&0&{\frac{1}{\mu + \sigma }}&0&0 \\ {\frac{{\alpha {A_5}}}{{\mu \left( {\alpha + \mu } \right)}} \, }&{\frac{\delta }{\mu (\delta + \mu )}}&{\frac{{\sigma {A_2}\left( {\alpha + \mu } \right)}}{\mu M}}&{\frac{1}{\mu }}&0 \\ { - \frac{{\alpha \mathop {\beta (t)}\limits^\sim {A_1}{A_6}}}{\mu M}}&{ - \frac{{\mathop {\beta (t)}\limits^\sim {A_1}}}{\mu (\delta + \mu )}}&{ - \frac{{\mathop {\beta (t)}\limits^\sim {A_1}{A_3}\left( {\alpha + \mu } \right)}}{\mu M}}&0&{\frac{1}{\mu }} \end{array}} \right).$$

where,

$$M = (\alpha + \mu )(\delta + \mu )(\sigma + \mu ),$$

$${A_1} = \gamma q - q + 1,$$

$${A_2} = \delta + \mu - \eta \mu ,$$

$${A_3} = \delta \rho + \eta \sigma + \mu p,$$

$${A_4} = \eta \sigma + \mu p + p\sigma - \eta p\sigma ,$$

$${A_5} = \delta \rho + \mu \sigma + \delta \mu p - \eta \mu \sigma - \mu p\sigma + \eta \mu p\sigma ,$$

$${A_6} = \delta \rho + \eta \sigma + \mu p + \mu \rho - \delta p\rho - \eta p\sigma - \mu p\sigma .$$

Hence,

$${R_0} = \rho \left( {F{V^{ - 1}}} \right) = \frac{{\mathop {\beta (t)}\limits^\sim {A_1}(\sigma \eta \alpha (1 - p) + \alpha p(\sigma + \mu ))}}{(\alpha + \mu )(\delta + \mu )(\sigma + \mu )} + \frac{{\mathop {\beta (t)}\limits^\sim \rho \alpha (1 - p){A_1}}}{(\alpha + \mu )(\sigma + \mu )}.$$

## Abbreviations

MuV: Mumps virus
MMR: Measles-Mumps-Rubella
DLMN: Distributed Lag Nonlinear Model
LHS: Latin hypercube sampling
AIC: Akaike Information Criterion
AICc: Akaike Information Criterion corrected
BIC: Bayesian Information Criterion
SSE: Sum of Squared Errors
SA: Sensitivity analysis
PRCC: Partial Rank Correlation Coefficient
WHO: World Health Organization
AM: Arithmetic Mean
SD: Standard Deviation
