# Supplementary material for: The impact of temperature, humidity and closing school on the mumps epidemic: a case study in the mainland of China
Source: BMC Public Health. 2024 Jun 19;24:1632. doi: 10.1186/s12889-024-18819-w (PMC11186224; doi:10.1186/s12889-024-18819-w)
Supplement: Supplementary file 1 — Supplementary Material 1. [file 12889_2024_18819_MOESM1_ESM.pdf]

Supplementary material

Xiaoqun Li* [1], Lianyun Zhang† [1], Changlei Tan[2], Yan Wu[3], Ziheng Zhang[4], Juan Ding‡[5], and Yong Li§[1]

[1]School of Information and Mathematics, Yangtze University, Jingzhou,434023, China
[2]Information Engineering College, Hunan Applied Technology University, Changde, 415100, China
[3]College of Applied Sciences, Beijing University of Technology, Beijing 100124, China
[4]School of Environment, Education & Development (SEED), The University of Manchester, Manchester M139PL, The United Kingdom
[5]Jingzhou Hospital Affiliated to Yangtze University, Jingzhou, 434023, Hubei, China

November 29, 2023

1 Fitting effect plot for each province

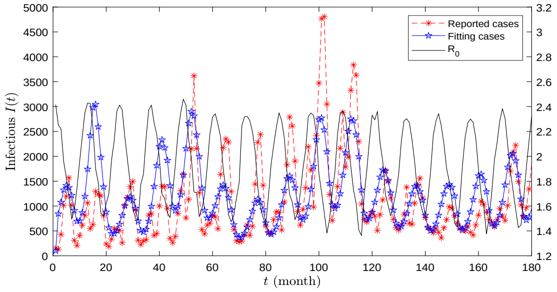

Figure 1: Anhui

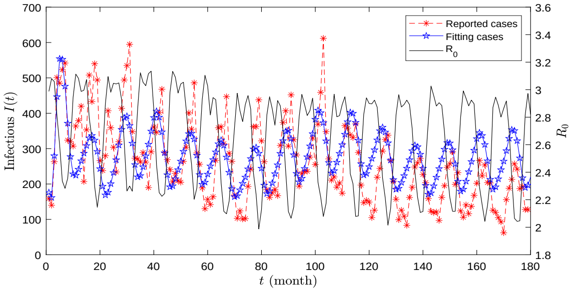

Figure 2: Beijing

*E-mail: 3010540792@qq.com
†E-mail: lianyun_zhang@163.com; Xiaoqun Li and Lianyun Zhang contributed equally to this work
‡E-mail: 308722048@qq.com; Corresponding author.
§E-mail: yongli@yangtzeu.edu.cn; Corresponding author.

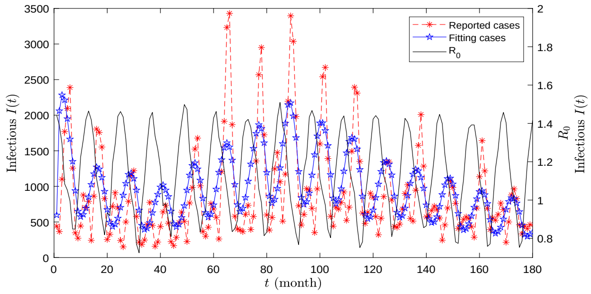

Figure 3: Chongqing

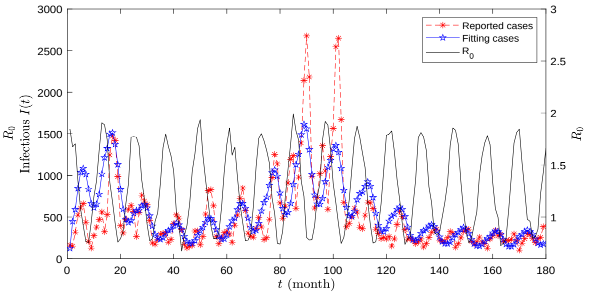

Figure 4: Fujian

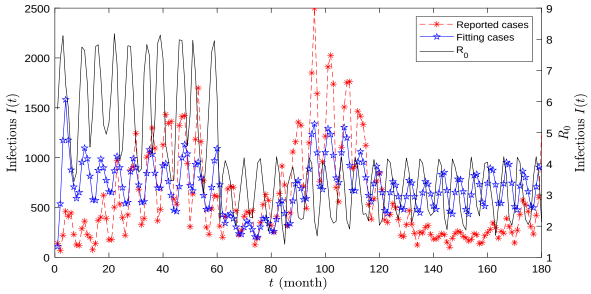

Figure 5: Gansu

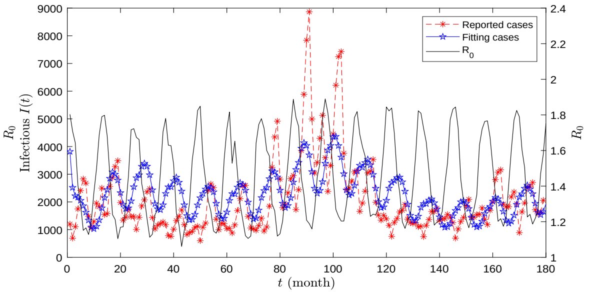

Figure 6: Guangdong

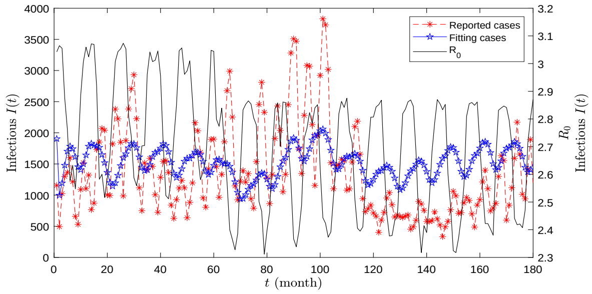

Figure 7: Guangxi

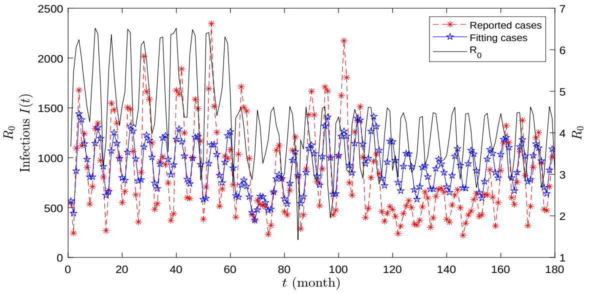

Figure 8: Guizhou

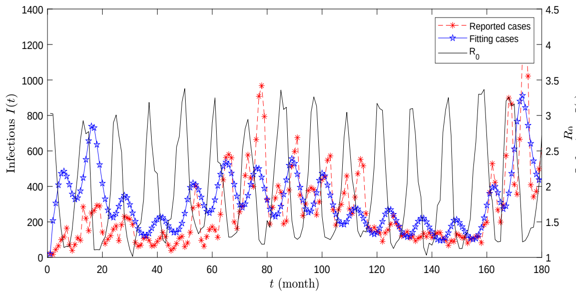

Figure 9: Hainan

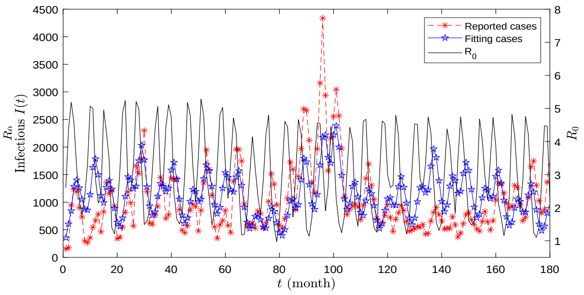

Figure 10: Hebei

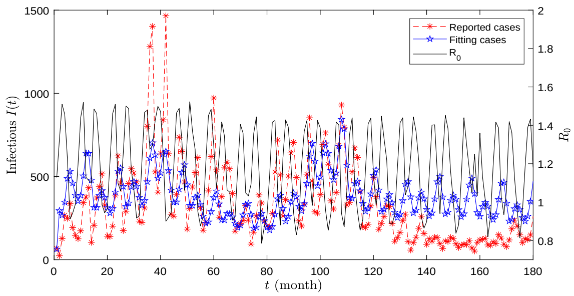

Figure 11: Heilongjiang

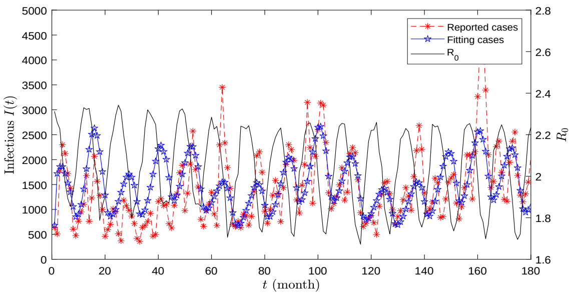

Figure 12: Henan

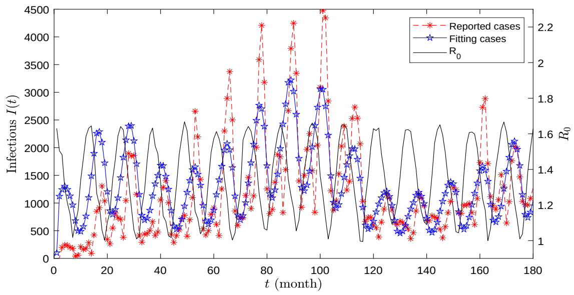

Figure 13: Hubei

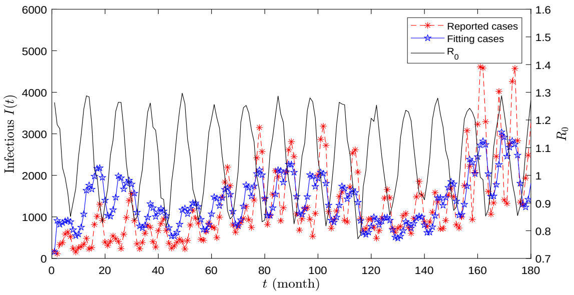

Figure 14: Hunan

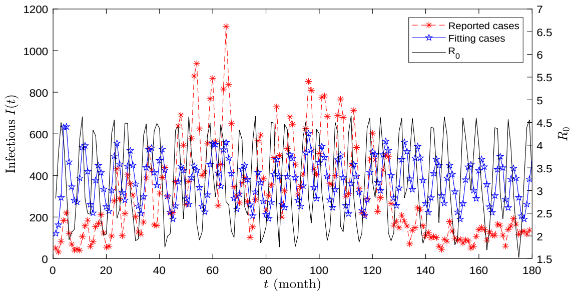

Figure 15: Jilin

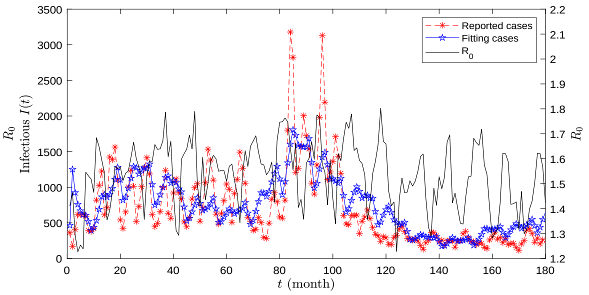

Figure 16: Liaoning

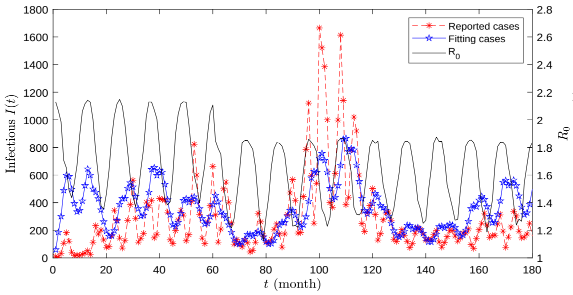

Figure 17: Inner Mongolia

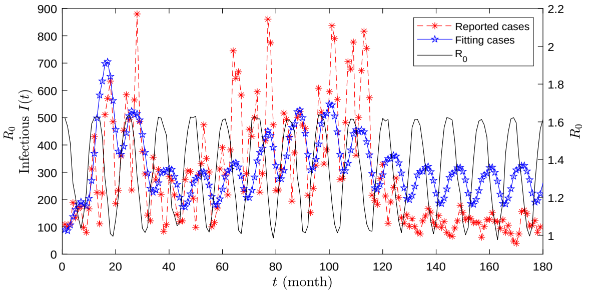

Figure 18: Ningxia

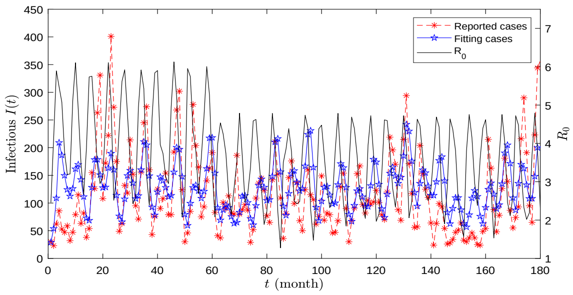

Figure 19: Qinghai

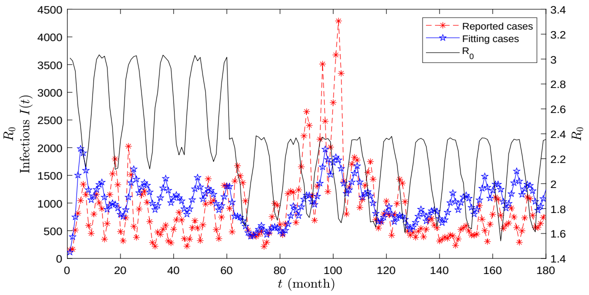

Figure 20: Shaanxi

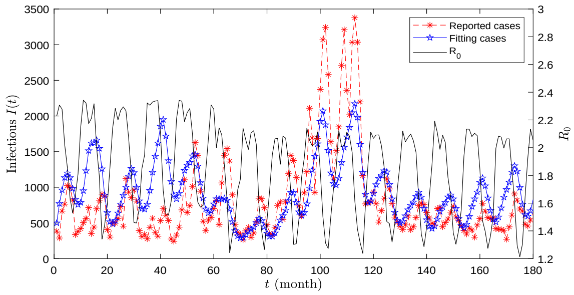

Figure 21: Shandong

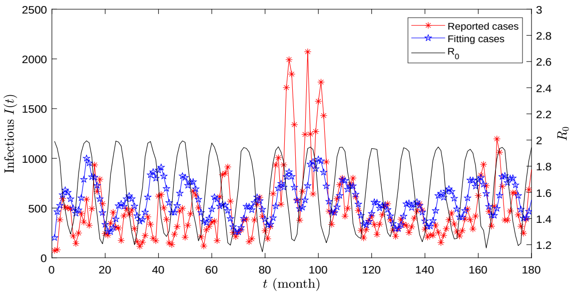

Figure 22: Shanxi

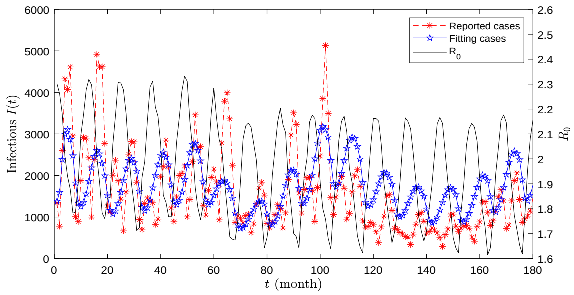

Figure 23: Sichuan

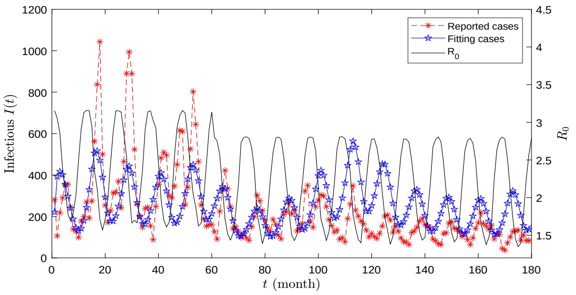

Figure 24: Tianjin

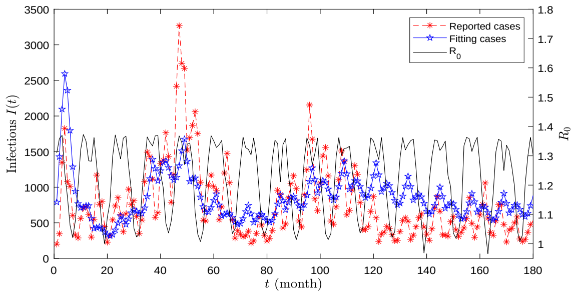

Figure 25: Xinjiang

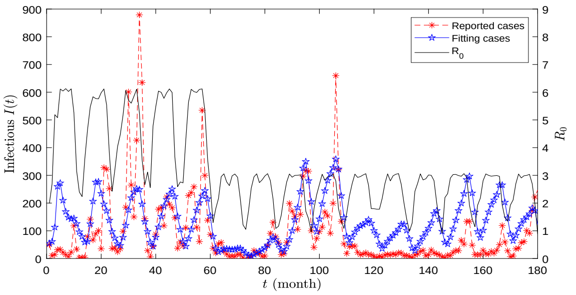

Figure 26: Tibet

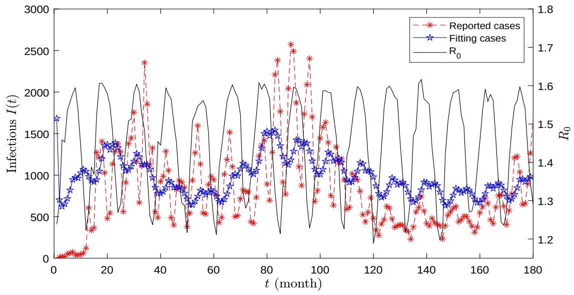

Figure 27: Yunnan

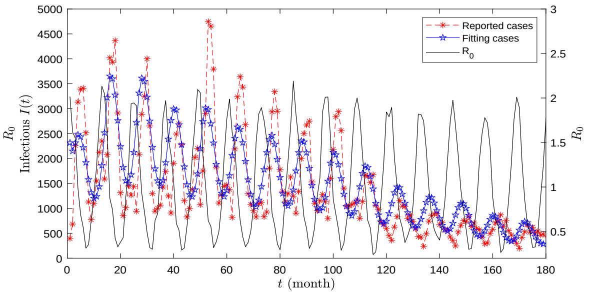

Figure 28: Zhejiang

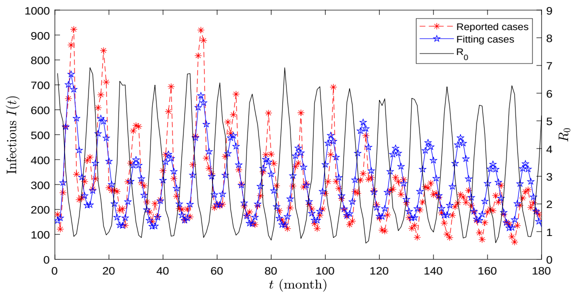

Figure 29: Shanghai

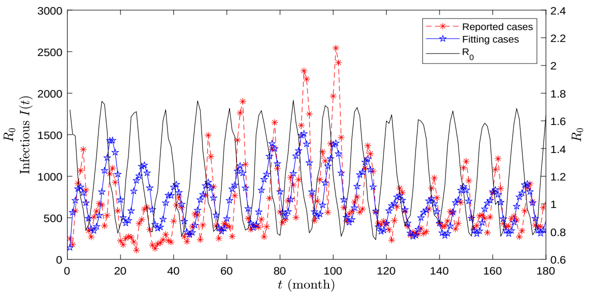

Figure 30: Jiangxi

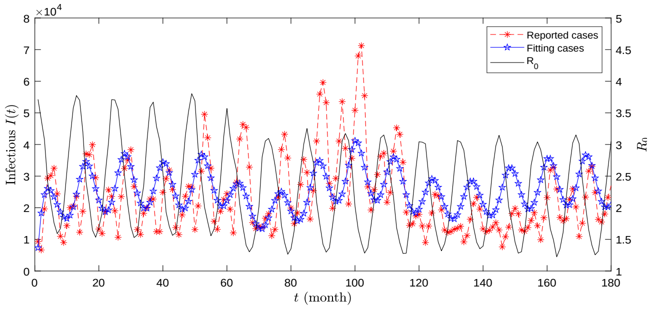

Figure 31: Nationwide

2 Temperature, humidity, school opening and closing, and the three factors combined to consider the influence of mumps in the graphs.

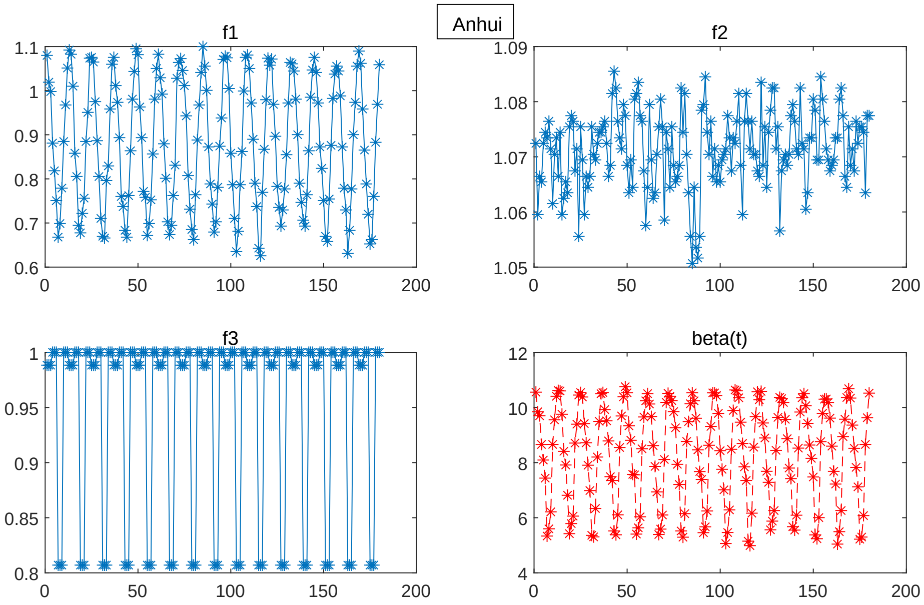

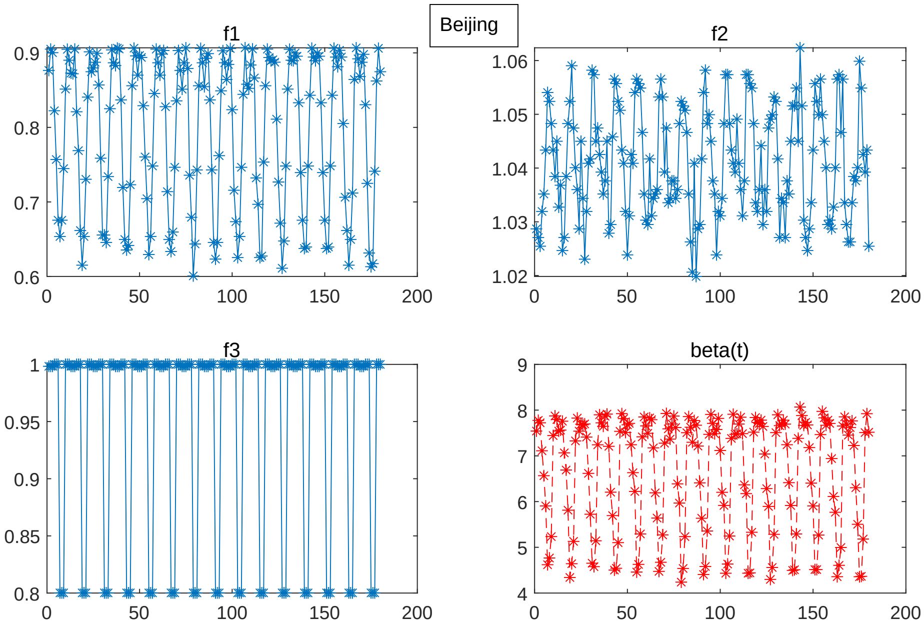

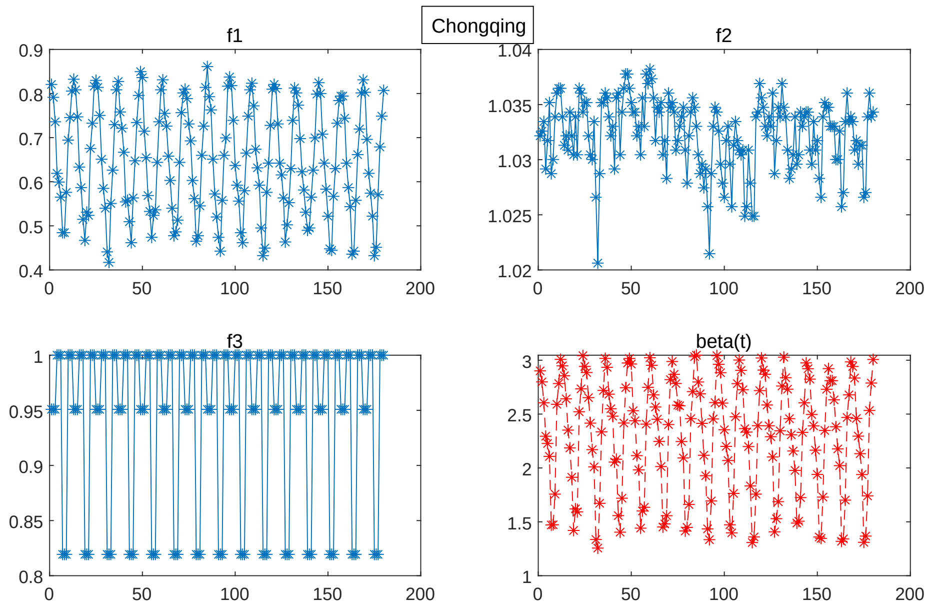
Chongqing
f1
f2
f3
beta(t)

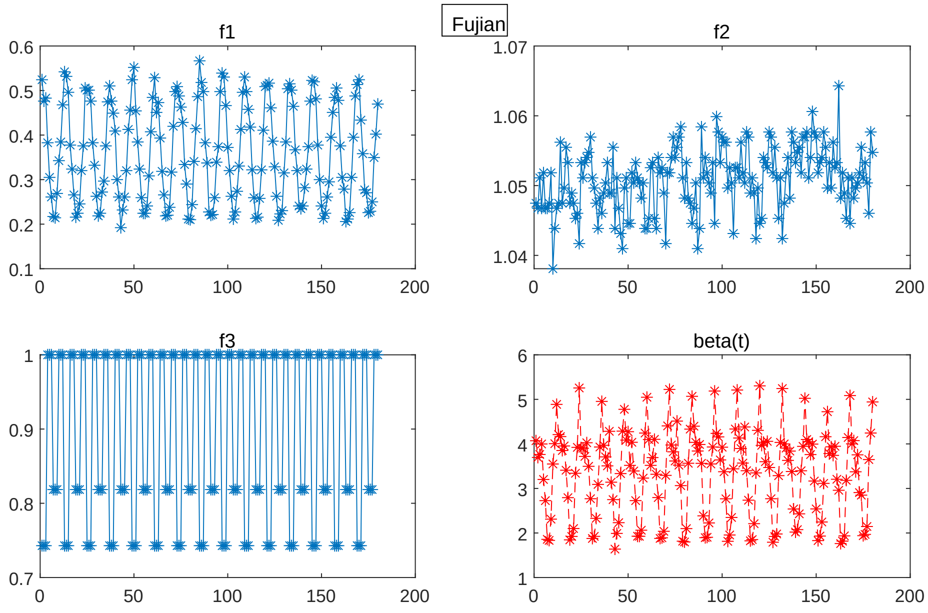
Fujian
f1
f2
f3
beta(t)

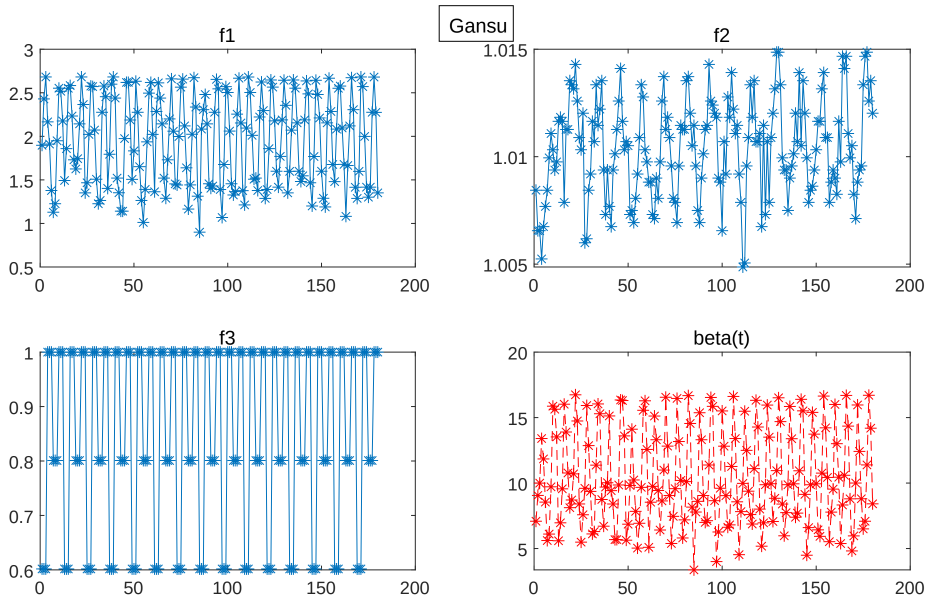
Gansu
f1
f2
f3
beta(t)

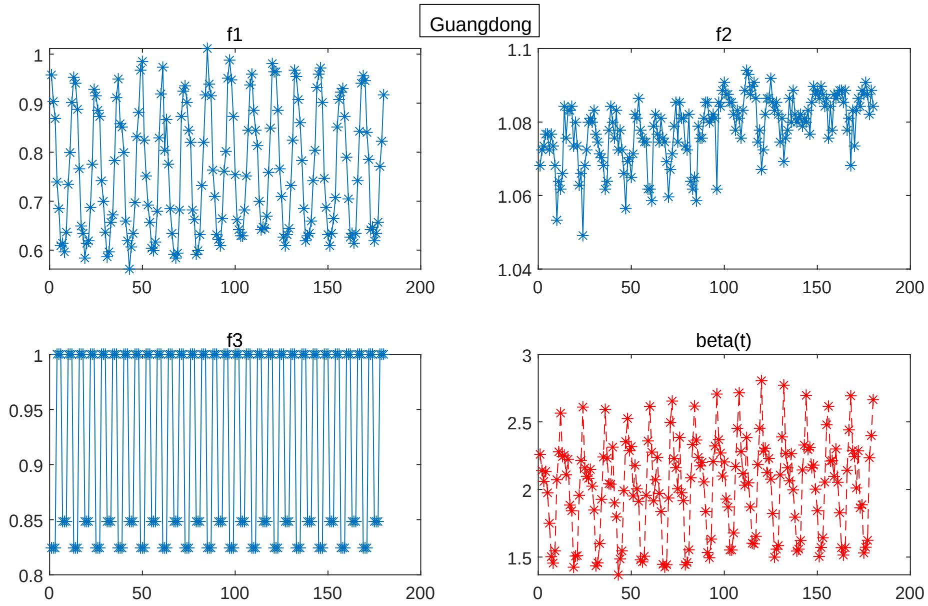
Guangdong
f1
f2
f3
beta(t)

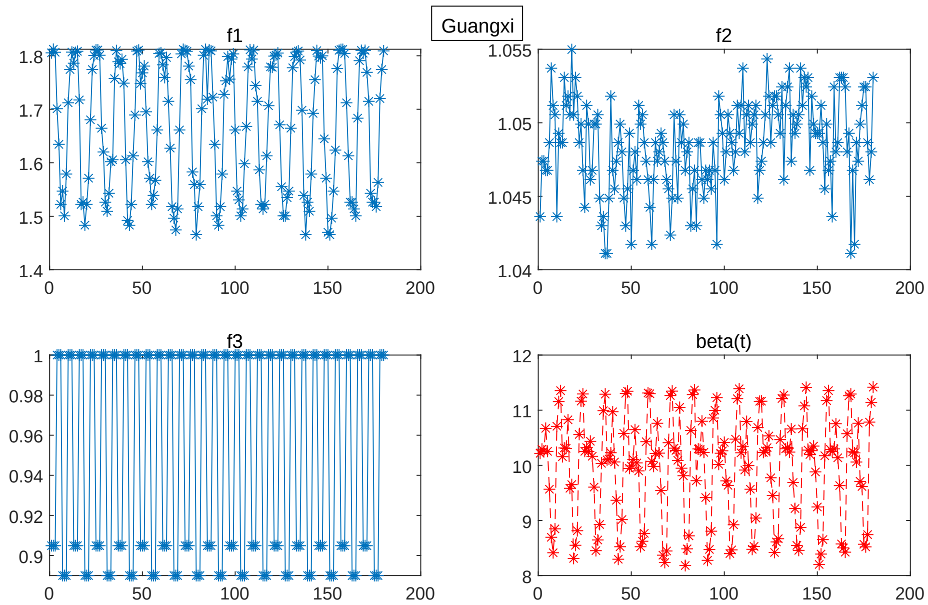
Guangxi
f1
f2
f3
beta(t)

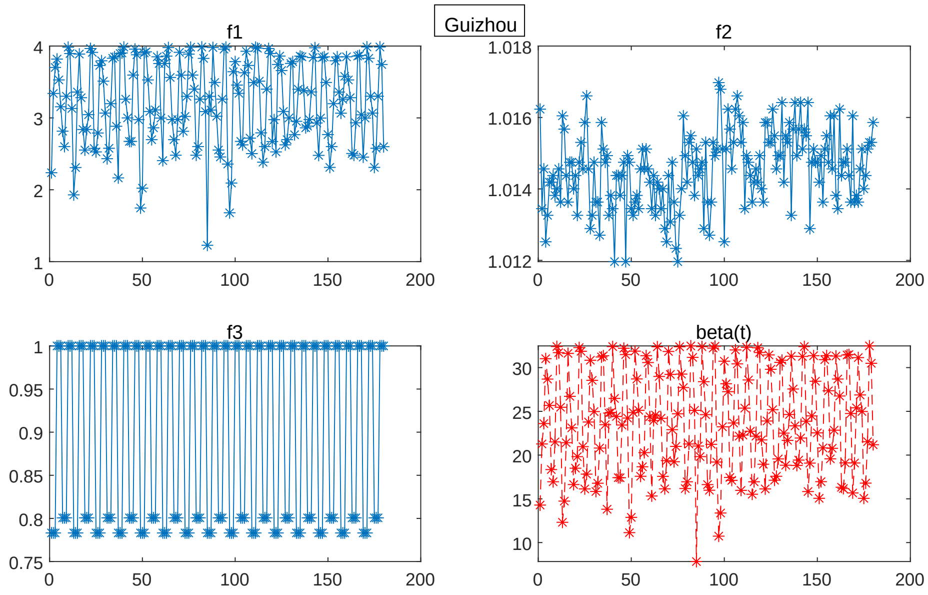
Guizhou
f1
f2
f3
beta(t)

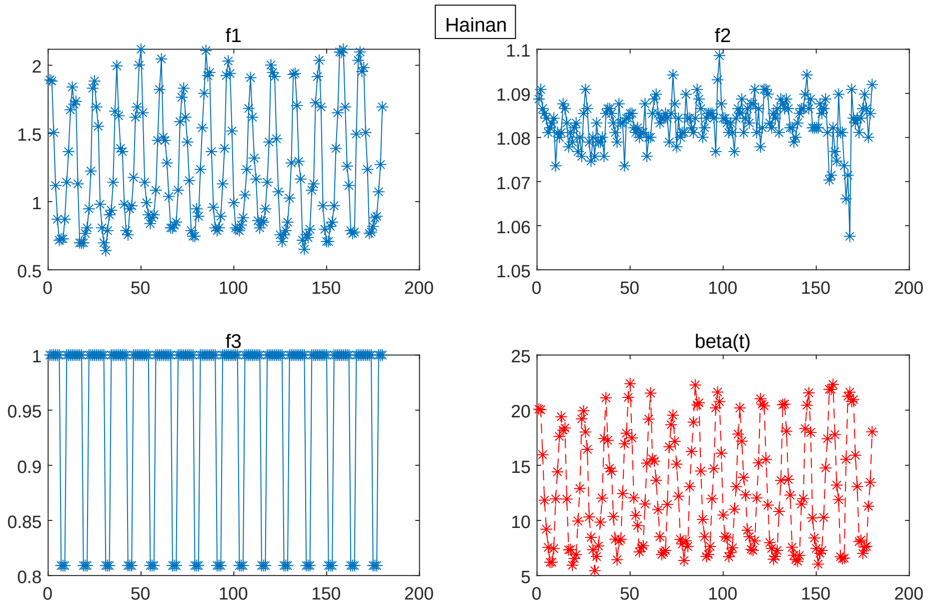
Hainan
f1
f2
f3
beta(t)

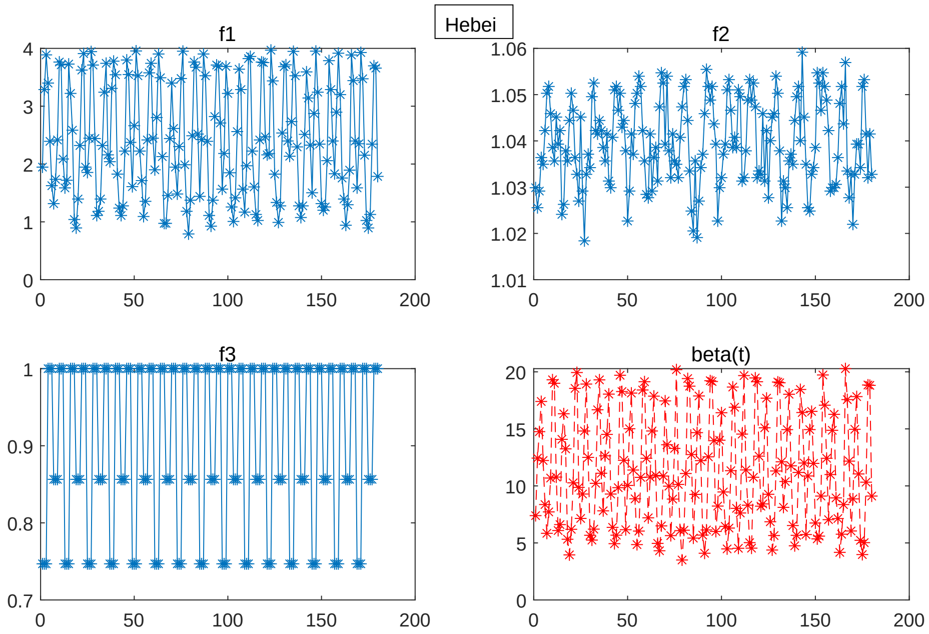
Hebei
f1
f2
f3
beta(t)

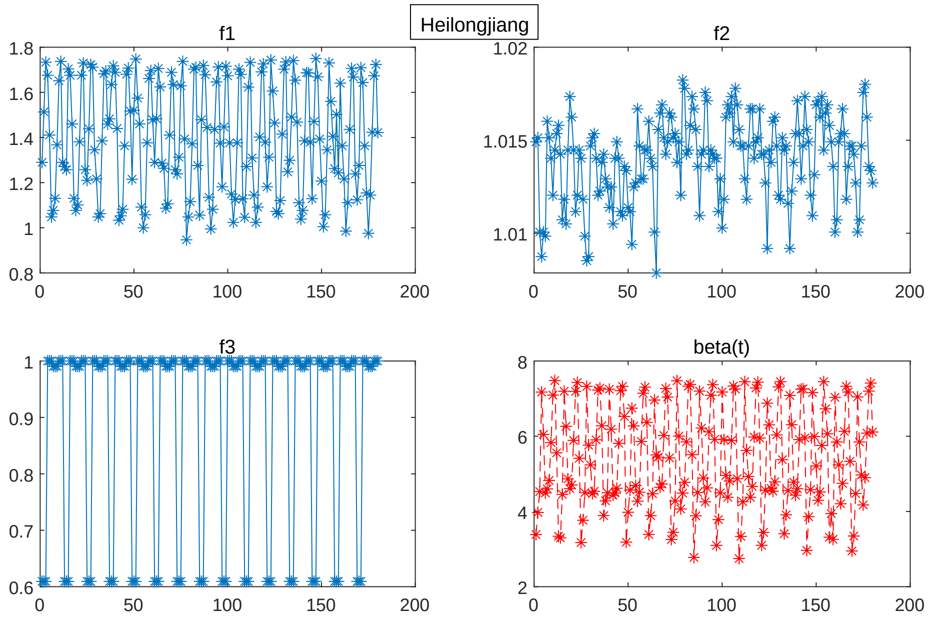
Heilongjiang
f1
f2
f3
beta(t)
1.8
1.6
1.4
1.2
1
0.8
1.02
1.015
1.01
0.9
0.7
0.6
8
6
4
2
0
50
100
150
200

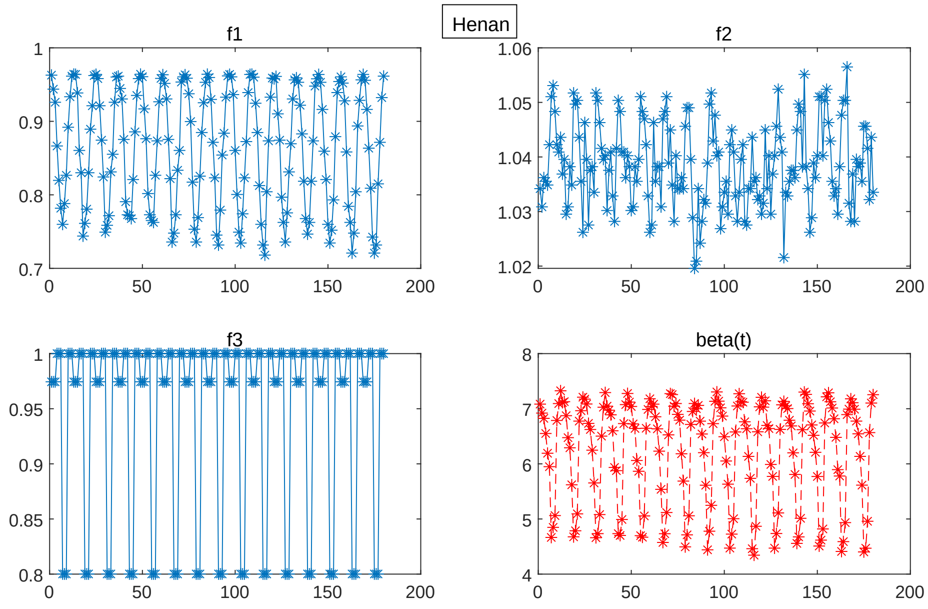
Henan
f1
f2
f3
beta(t)
1
0.9
0.8
0.7
1.06
1.05
1.04
1.03
1.02
0.95
0.85
8
7
6
5
4
0
50
100
150
200

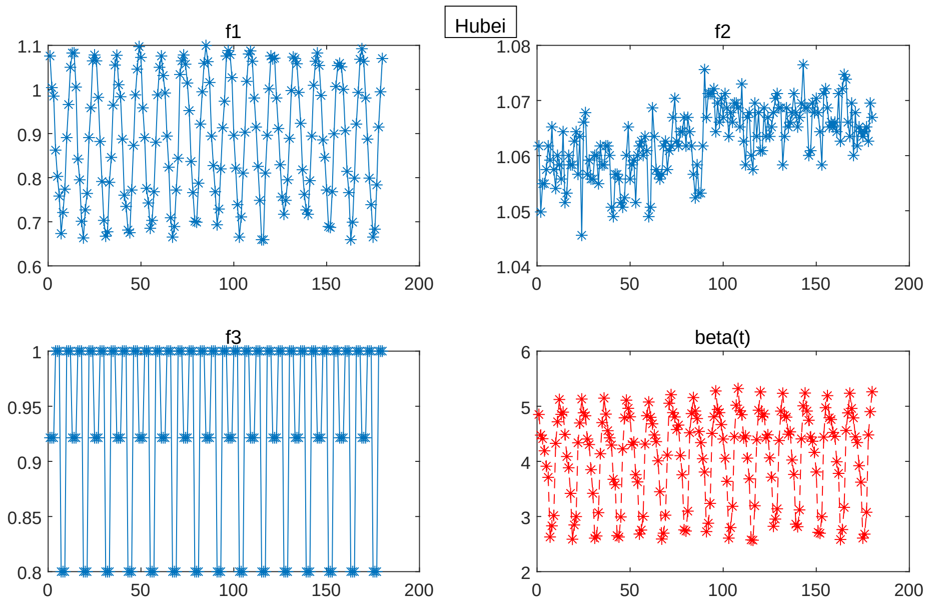
Hubei
f1
f2
f3
beta(t)

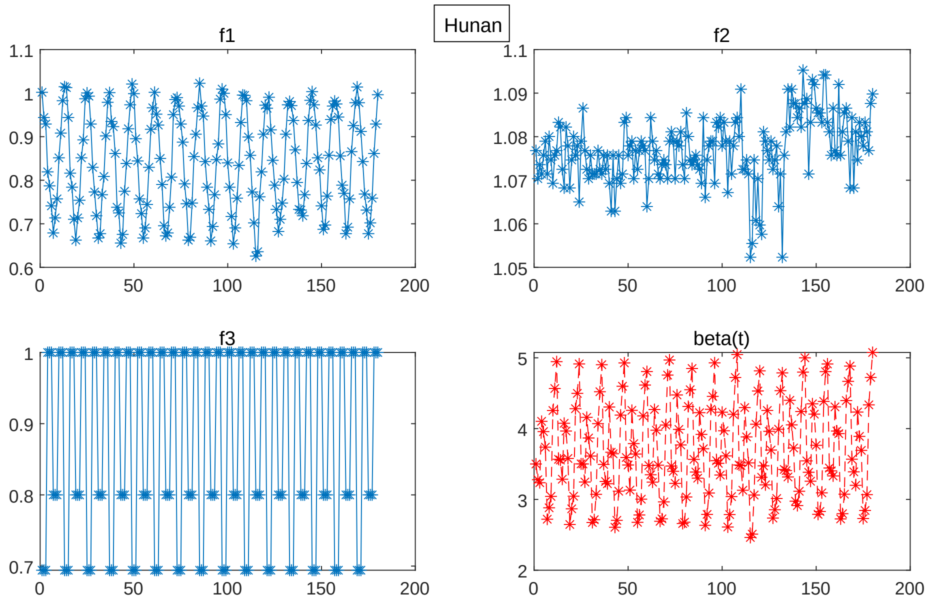
Hunan
f1
f2
f3
beta(t)

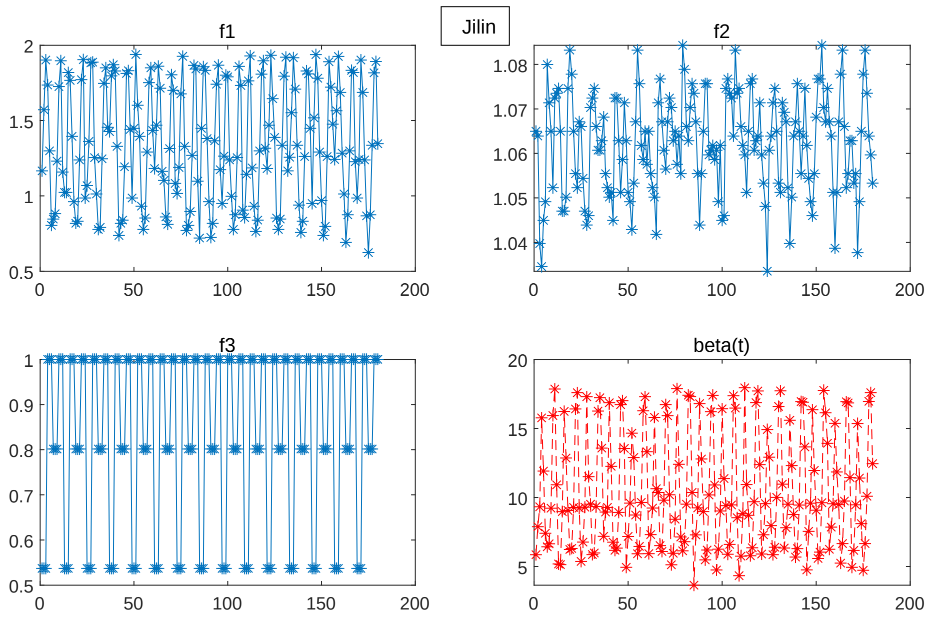
Jilin
f1
f2
f3
beta(t)
0
50
100
150
200

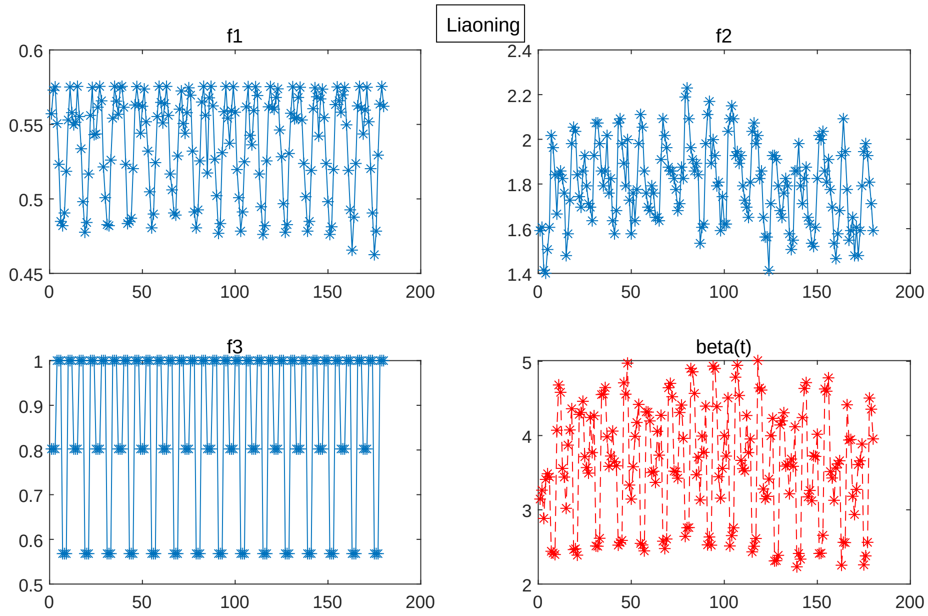
Liaoning
f1
f2
f3
beta(t)
0
50
100
150
200

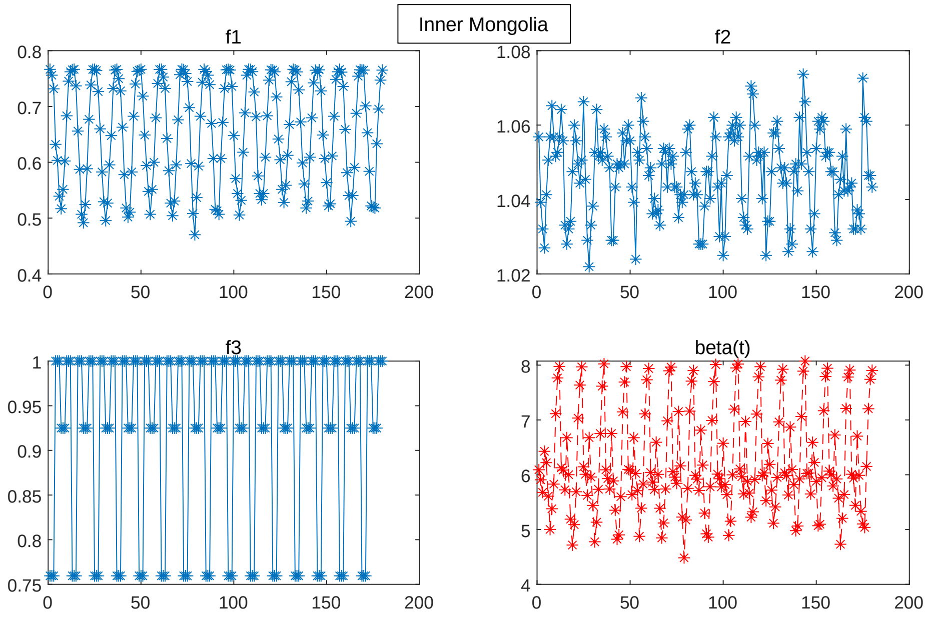
Inner Mongolia
f1
f2
f3
beta(t)

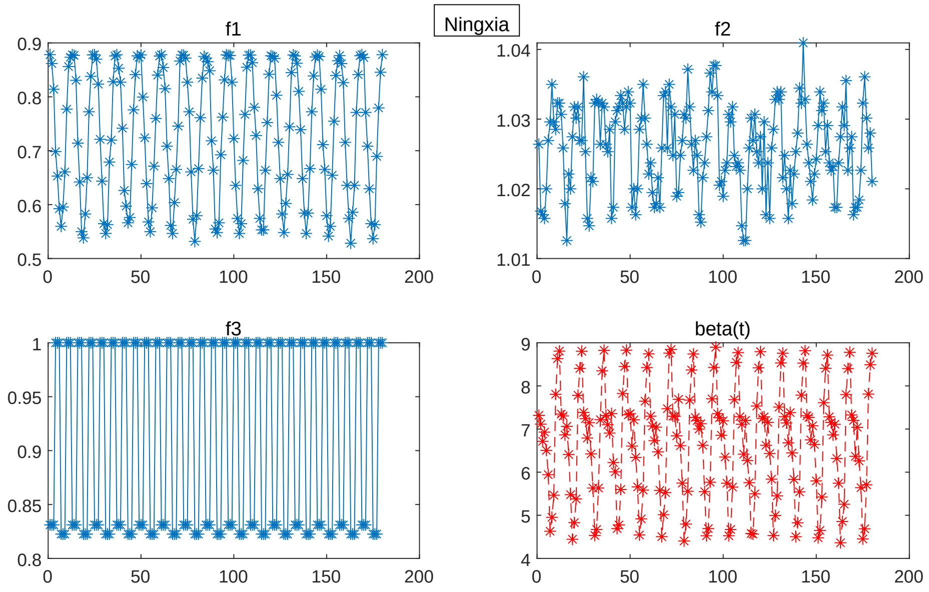
Ningxia
f1
f2
f3
beta(t)

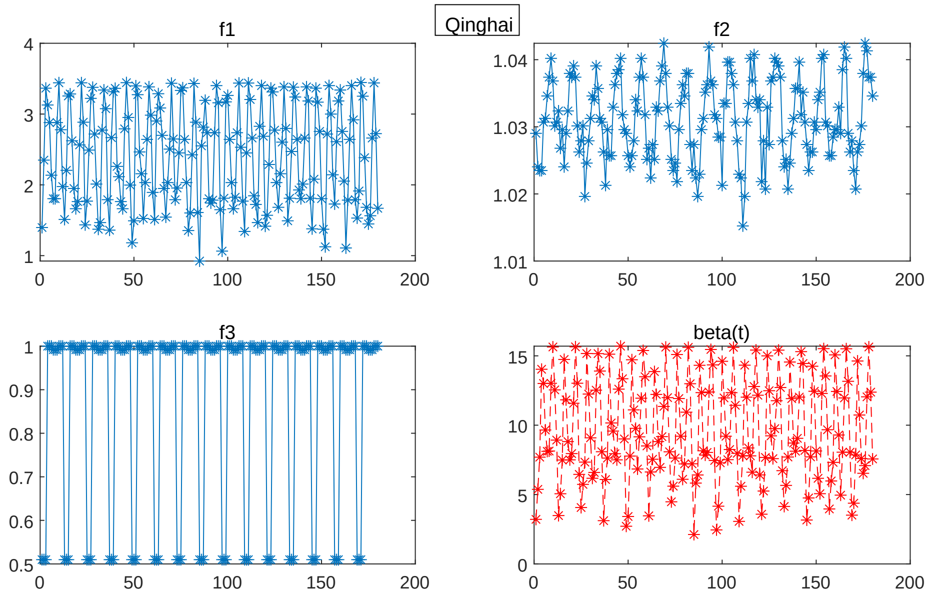
Qinghai
f1
f2
f3
beta(t)

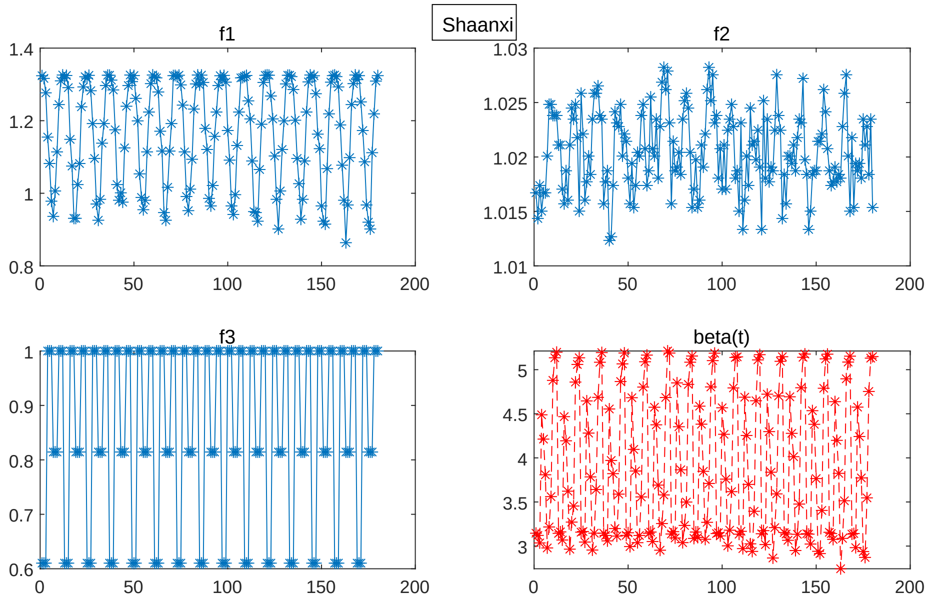
Shaanxi
f1
f2
f3
beta(t)

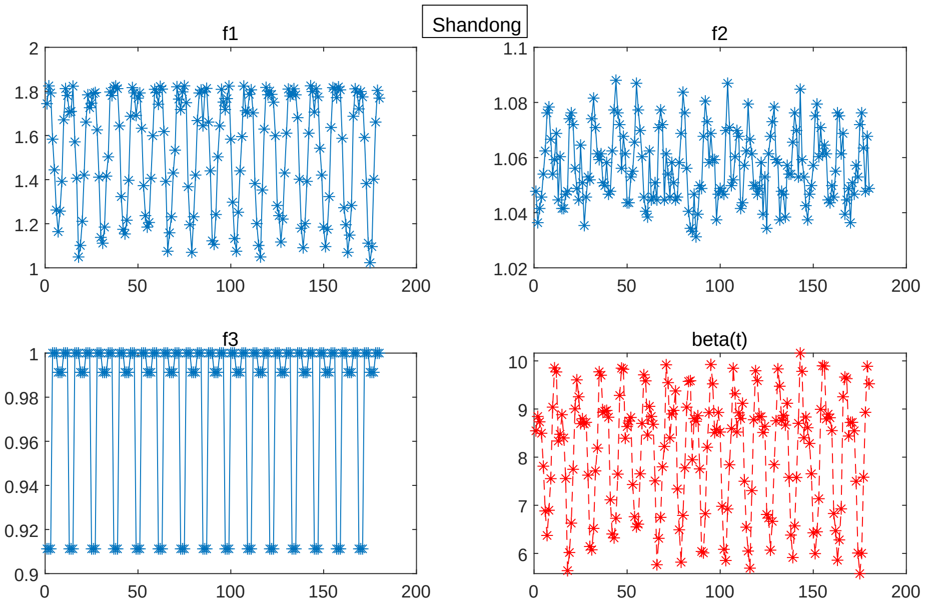
Shandong
f1
2
1.8
1.6
1.4
1.2
1
0
50
100
150
200
f2
1.1
1.08
1.06
1.04
1.02
0
50
100
150
200
f3
1
0.98
0.96
0.94
0.92
0.9
0
50
100
150
200
beta(t)
10
9
8
7
6
0
50
100
150
200

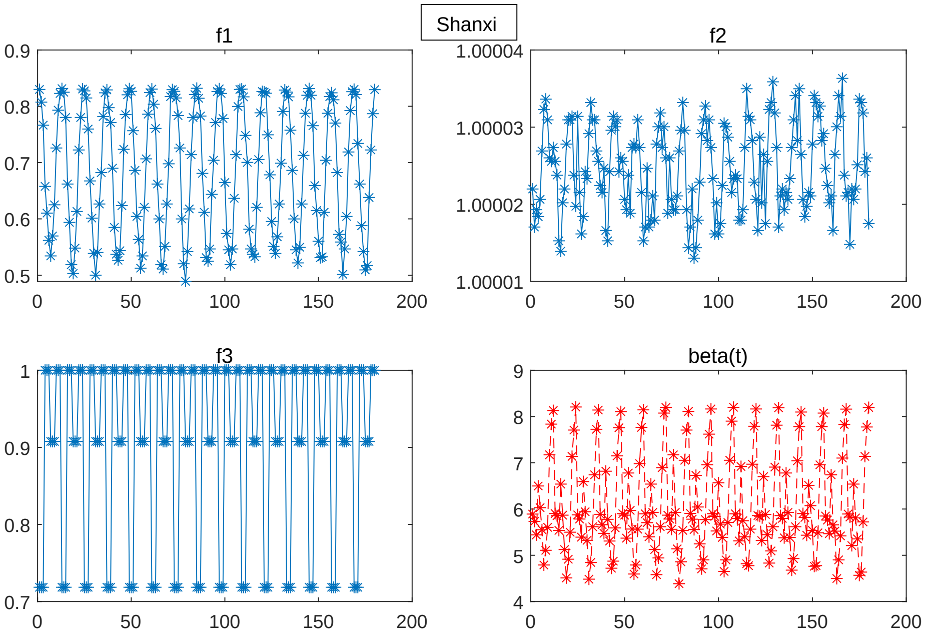
Shanxi
f1
0.9
0.8
0.7
0.6
0.5
0
50
100
150
200
f2
1.00004
1.00003
1.00002
1.00001
0
50
100
150
200
f3
1
0.9
0.8
0.7
0
50
100
150
200
beta(t)
9
8
7
6
5
4
0
50
100
150
200

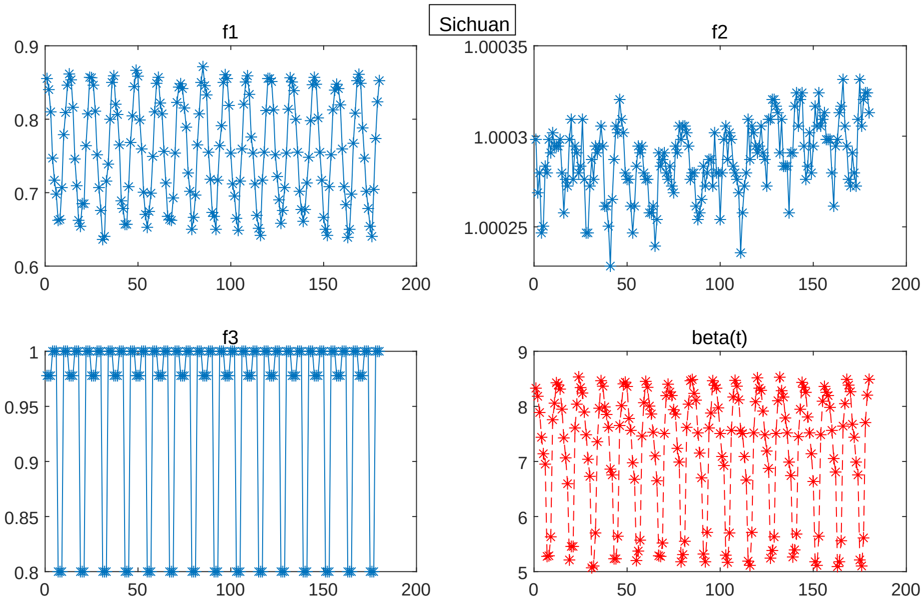
Sichuan
f1
f2
f3
beta(t)
0.9
0.8
0.7
0.6
1.00035
1.0003
1.00025
1
0.95
0.9
0.85
0.8
9
8
7
6
5
0
50
100
150
200

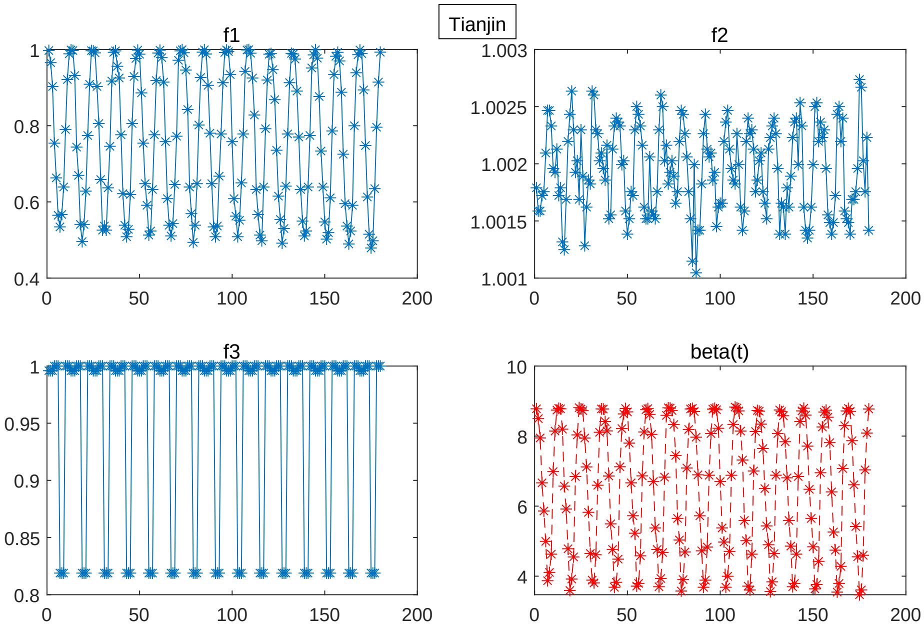
Tianjin
f1
f2
f3
beta(t)
1
0.8
0.6
0.4
1.003
1.0025
1.002
1.0015
1.001
1
0.95
0.9
0.85
0.8
10
8
6
4
0
50
100
150
200

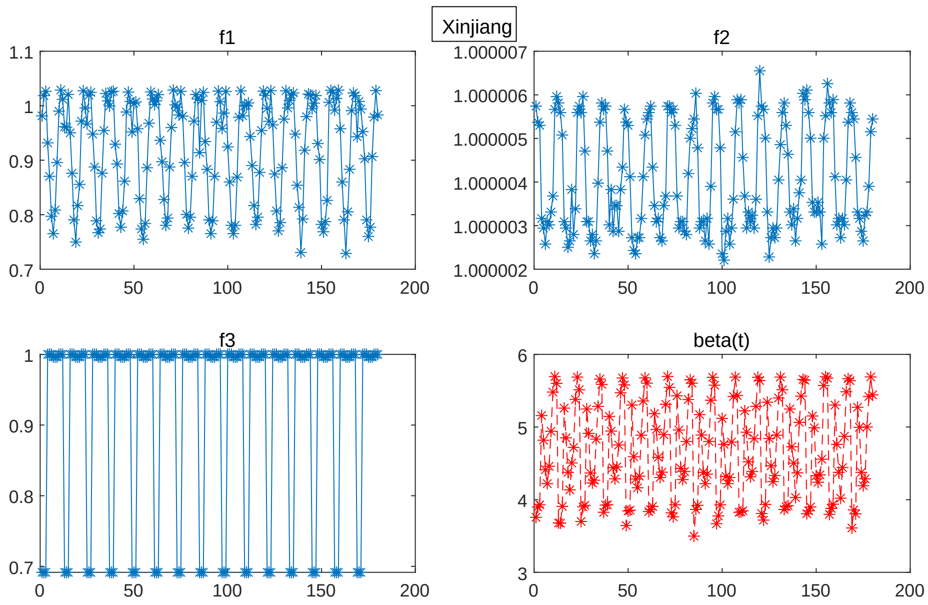
Xinjiang
f1
f2
f3
beta(t)

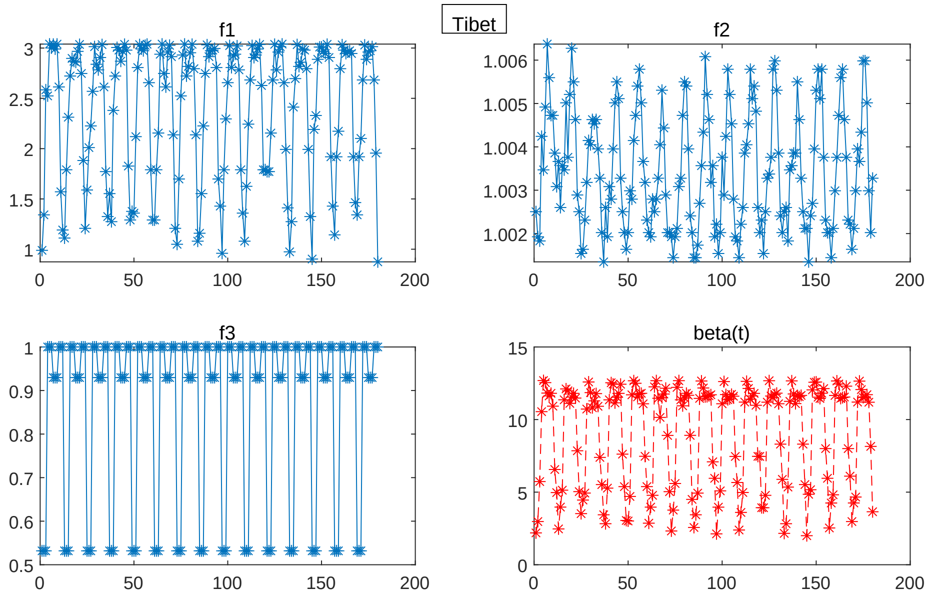
Tibet
f1
f2
f3
beta(t)

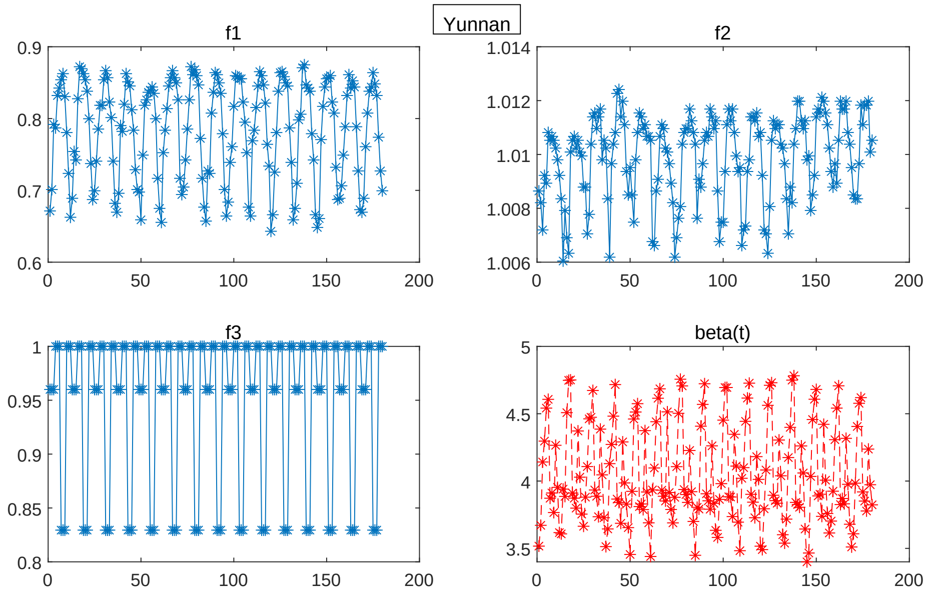
Yunnan
f1
f2
f3
beta(t)
0.9
0.8
0.7
0.6
1.014
1.012
1.01
1.008
1.006
1
0.95
0.9
0.85
0.8
5
4.5
4
3.5
0
50
100
150
200

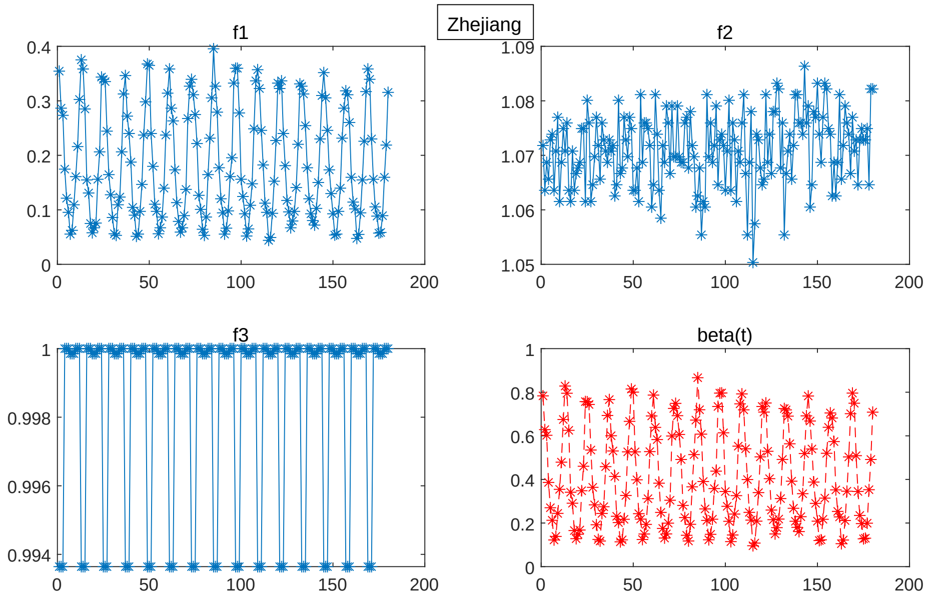
Zhejiang
f1
f2
f3
beta(t)
0.4
0.3
0.2
0.1
0
1.09
1.08
1.07
1.06
1.05
1
0.998
0.996
0.994
1
0.8
0.6
0.4
0.2
0
0
50
100
150
200

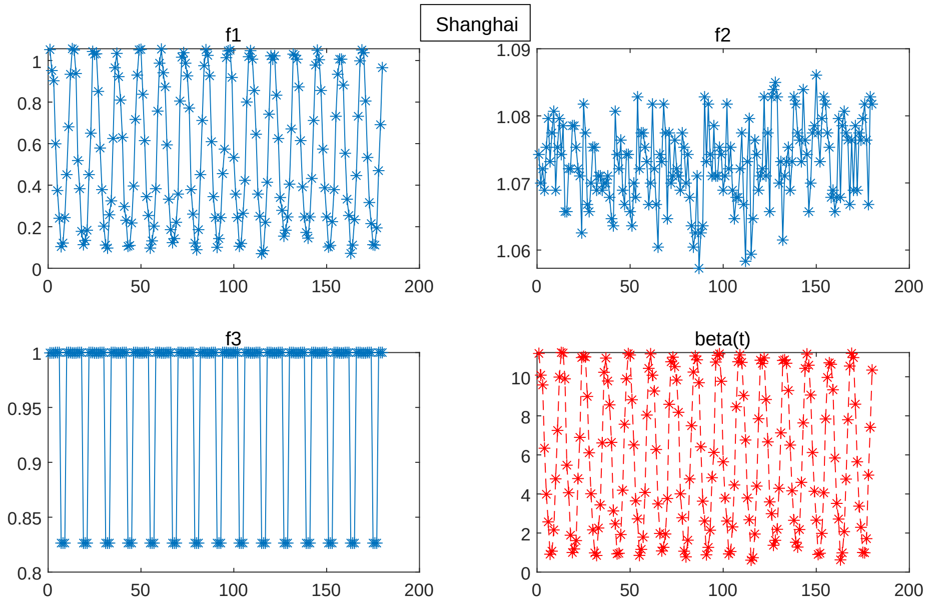
Shanghai
f1
f2
f3
beta(t)

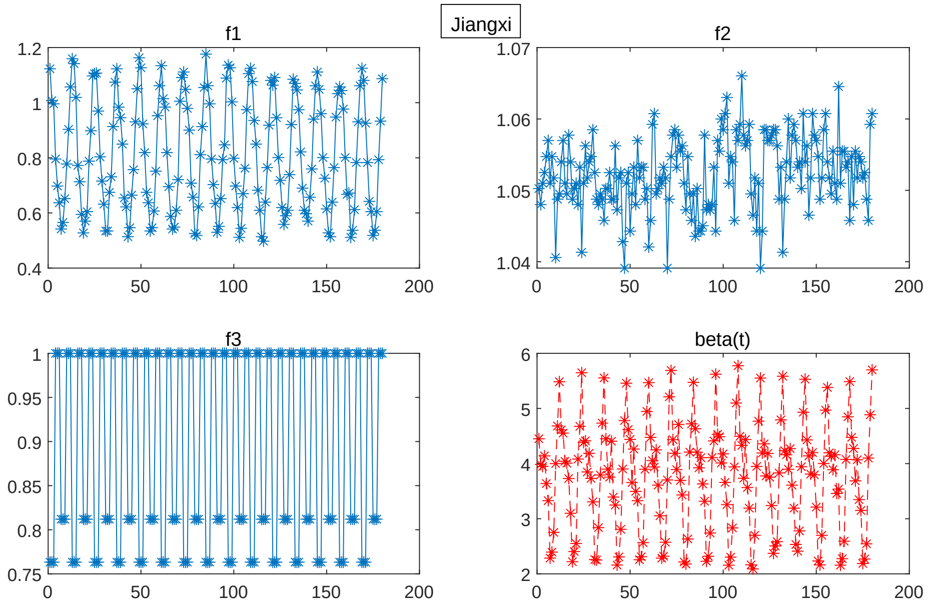
Jiangxi
f1
f2
f3
beta(t)

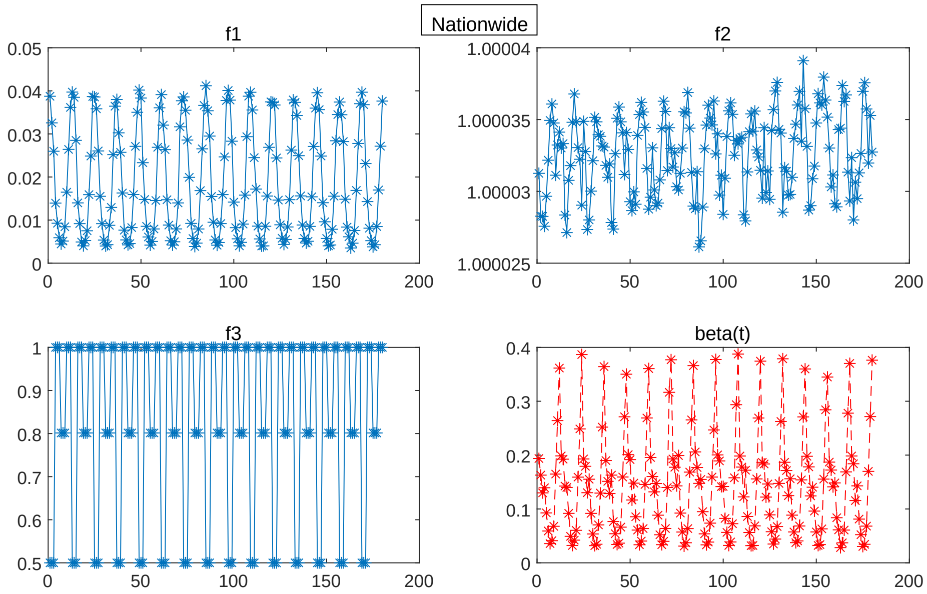
Nationwide
f1
0.05
0.04
0.03
0.02
0.01
0
0
50
100
150
200
f2
1.00004
1.000035
1.00003
1.000025
0
50
100
150
200
f3
1
0.9
0.8
0.7
0.6
0.5
0
50
100
150
200
beta(t)
0.4
0.3
0.2
0.1
0
0
50
100
150
200
